# Sbno2-mediated tissue-resident alveolar macrophages: a novel therapeutic axis for sepsis-induced acute lung injury

**DOI:** 10.1038/s41420-025-02772-7

**Published:** 2026-01-05

**Authors:** Jingyu Dai, Zhihai Wu, Jiayi Zhong, Xiaolong Wu, Yibin Liu, Qin Yang, Li Li, Shuyao Zhang, Junyong Zhong

**Affiliations:** 1 Oncology Department, Longgang Central Hospital of Shenzhen, Shenzhen, Guangdong PR China; 2 Shantou University Medical College, Shantou, Guangdong PR China; 3 Department of Pharmacy, Guangzhou Red Cross Hospital of Jinan University, Guangzhou, PR China

**Keywords:** Respiratory tract diseases, Molecular biology

## Abstract

Sepsis-induced acute lung injury (ALI) is a critical clinical condition characterized by severe inflammation and alveolar epithelial barrier disruption, with limited effective treatments. Our study investigates the role of Sbno2-expressing tissue-resident alveolar macrophages (TR-AMs) in promoting alveolar epithelial cell (AEC) regeneration and barrier function in sepsis-induced ALI. Utilizing single-cell RNA sequencing (scRNA-seq), we identified significant upregulation of Sbno2 in TR-AMs, which correlated with enhanced AEC proliferation and reduced apoptosis. Functional assays demonstrated that Sbno2-expressing TR-AMs substantially supported alveolar structure regeneration in both in vitro and in vivo models. Knockout of Sbno2 in TR-AMs impaired AEC proliferation and compromised lung barrier integrity. Therapeutic administration of recombinant Sbno2 (rSbno2) in a sepsis-induced ALI mouse model alleviated lung injury, promoted AEC proliferation, and restored barrier function, highlighting Sbno2 as a potential therapeutic target for ALI. These findings provide novel insights into the molecular mechanisms of lung repair in sepsis-induced ALI and suggest that enhancing Sbno2 expression in TR-AMs could be a promising strategy for improving outcomes in patients with ALI.

## Facts

- The Sbno2 gene is significantly upregulated in tissue-resident alveolar macrophages (TR-AMs) and correlates with enhanced proliferation of alveolar epithelial cells (AECs).
- Sbno2 expression plays a critical role in maintaining alveolar barrier integrity and significantly reduces inflammation in acute lung injury (ALI).
- In the LPS-induced ALI model, exogenous Sbno2 (rSbno2) treatment substantially promotes AEC regeneration and tissue repair.
- TR-AMs mediate both inflammation regulation and tissue regeneration during the recovery phase of ALI.
- Single-cell transcriptomics reveals key molecular mechanisms underlying TR-AM and AEC interactions in tissue repair.

## Open Questions

- What are the precise molecular mechanisms by which Sbno2 regulates AEC proliferation and barrier function?
- Does Sbno2 influence other immune or non-immune cells involved in lung tissue repair?
- Is Sbno2’s role consistent across different pathological conditions, such as pulmonary fibrosis or chronic obstructive pulmonary disease (COPD)?
- What is the clinical translational potential and safety profile of rSbno2 therapy?
- Are there other cell subtypes beyond TR-AMs that regulate Sbno2 expression and function?

## Introduction

Acute Lung Injury (ALI) is a severe complication often caused by sepsis, with complex pathogenesis and limited therapeutic strategies [1]. Within the respiratory system, alveoli serve as the primary site for gas exchange, with their epithelial cells playing a crucial role in maintaining oxygen exchange function and lung barrier integrity [2, 3]. ALI patients typically exhibit progressive alveolar inflammation and disruption of the blood-gas barrier, leading to a rapid deterioration in respiratory function and serious consequences that significantly impact patient survival rates and quality of life [4, 5].

Alveolar Macrophages (AMs), as a vital immune cell population in the alveoli, play a critical role in maintaining lung immune homeostasis, eliminating pathogenic microorganisms, and regulating inflammatory responses [6–8]. In the lungs of conditional knockout mice, an imbalance in the immune and metabolic states of tissue-resident alveolar macrophages (TRAMs) leads to spontaneous inflammatory injury and respiratory diseases. STIMATE+ ADEs are absorbed by TRAMs, regulating high Ca2+ reactivity and prolonged Ca2+ signaling to maintain an M2-like immune phenotype and metabolic selection [9]. With ongoing research, the role of TR-AMs in lung injury and repair has become increasingly important, particularly drawing widespread attention to sepsis-induced ALI within the academic community [10, 11].

Previous studies have shown that Sbno2 acts as a novel negative feedback regulator of IL-6, capable of suppressing excessive inflammatory responses in the brain [12]. The two isoforms of Sbno2 drive distinct gene networks, with isoform 2 primarily influencing the antimicrobial activity of macrophages [13]. It has been found that IL-10 induces the expression of the ETS family transcriptional repressor ETV3 and the helicase family co-inhibitor strawberry notch homolog 2 (Sbno2) in mouse and human macrophages. ETV3 and Sbno2 are components of the IL-10 downstream pathway that contribute to its anti-inflammatory effects [14].

This study aims to delve into the mechanistic role of the Sbno2 gene in TR-AMs to elucidate its regulatory mechanisms in AEC regeneration and its specific role in sepsis-induced ALI. Through the application of single-cell RNA sequencing (scRNA-seq) technology and cell isolation experiments, we aim to comprehensively understand the relationship between TR-AMs with high Sbno2 expression and the regenerative capacity of lung epithelial cells, exploring the regulatory mechanisms of Sbno2 in this process. The purpose of this research is to deeply investigate the molecular mechanisms underlying pulmonary diseases, providing scientific basis and experimental support for the development of more effective ALI treatment strategies in the future.

This study aims to further investigate the mechanisms by which TR-AMs expressing Sbno2 promote alveolar epithelial cell (AEC) regeneration and delay ALI, providing theoretical foundations and scientific support for the clinical treatment of ALI. Through an in-depth exploration of this molecular mechanism, we hope to uncover new therapeutic targets and provide scientific evidence for developing more effective treatment strategies, thereby improving survival rates and outcomes for patients suffering from ALI induced by sepsis.

## Results

### LPS induces changes in multiple cell types and regulates functions in lung tissue in ALI

**Fig. 1:**
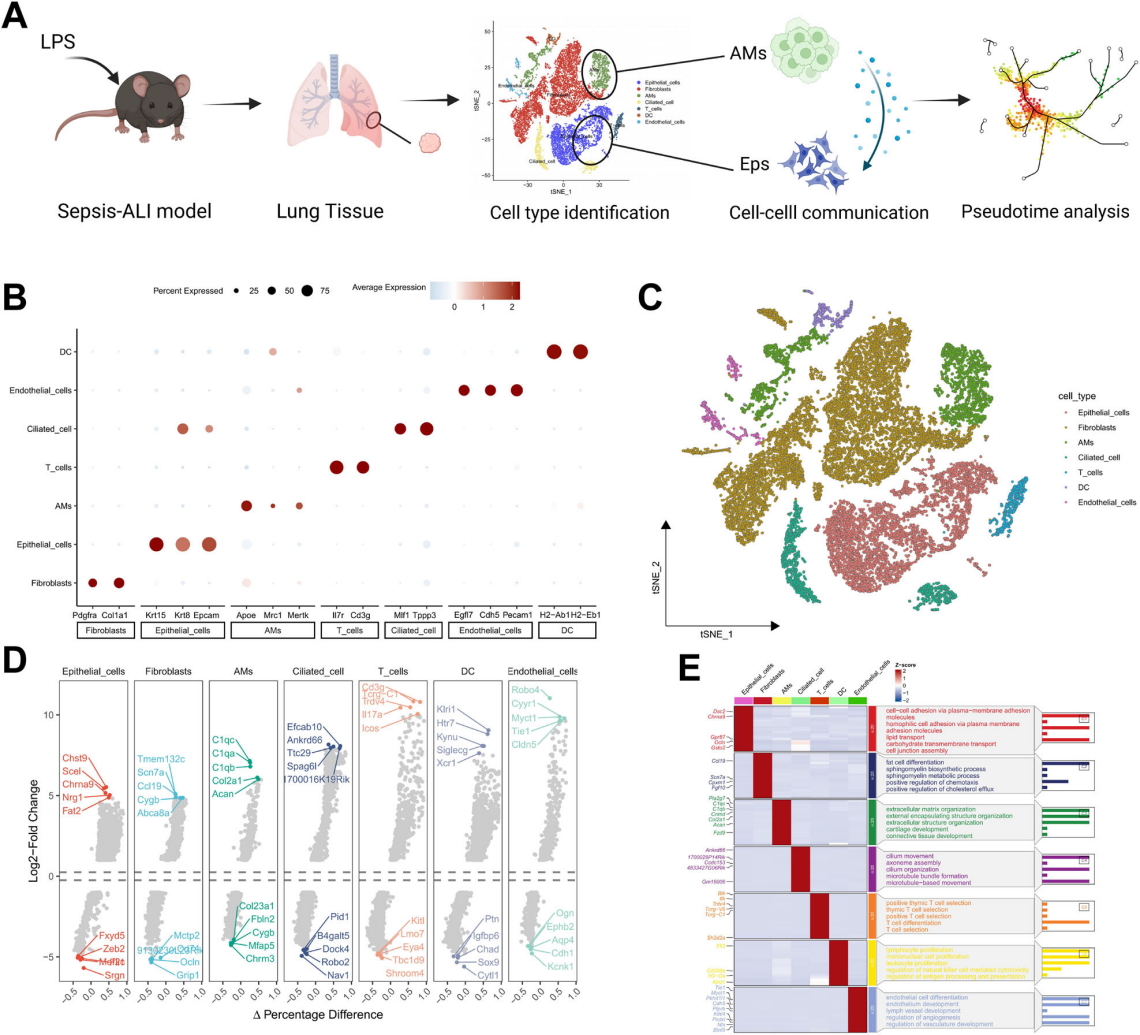
Single-cell landscape of lung tissue in LPS-induced Sepsis-ALI mice. **A** Workflow diagram of single-cell extraction from lung tissues of Naïve and LPS model mice using 10x Genomics technology (Created with BioRender.com); **B** Dot plot of cell type-specific marker gene expression. Red indicates high expression, blue indicates low expression, and circle size represents the percentage of cells expressing the gene; **C** Cell type annotation and clustering based on t-SNE; **D** Volcano plots of differentially expressed genes for each cell subtype (top: upregulated genes; bottom: downregulated genes); **E** Gene Ontology-biological process (GO-BP) enrichment heatmap. The left panel shows gene expression clustering; the right panel displays enriched biological pathways.

To investigate the cellular effects of LPS-induced sepsis-associated ALI on lung tissue, we performed scRNA-seq on lung tissues from one randomly selected PBS-injected mouse (Naïve control) and one LPS-induced sepsis-ALI mouse. The experimental design workflow is illustrated in Fig. 1A. Data integration was conducted using the Seurat package. Initially, we assessed the number of detected genes (nFeature_RNA), the number of mRNA molecules (nCount_RNA), and the percentage of mitochondrial genes (percent.mt) for all cells in the scRNA-seq dataset (Fig. S1A). Low-quality cells were filtered out using the criteria nFeature_RNA > 200, nCount_RNA < 100,000, and percent.mt < 20, resulting in an expression matrix comprising 32,285 genes and 22,970 cells.

Highly variable genes were selected based on expression variance, and the top 2000 most variable genes were used for downstream analysis. PCA was applied for linear dimensionality reduction, and the distribution of cells in PC_1 and PC_2 revealed minimal batch effects between samples (Fig. S1B). The optimal number of principal components was determined using an ElbowPlot, and the top 17 PCs were used for nonlinear dimensionality reduction via t-SNE (Fig. S1C, D). Clustering analysis identified 23 distinct clusters (Fig. S1E). Cell types were annotated based on known lineage-specific marker genes and the CellMarker online database (Figs. 1B and S1F), resulting in the identification of seven major cell types: epithelial cells, fibroblasts, AMs, ciliated cells, T cells, dendritic cells (DCs), and endothelial cells (Fig. 1C). The cellular composition of each sample is shown in Fig. S1G.

We then performed enrichment analysis on the differentially expressed genes among these seven cell types (Fig. 1D), revealing their involvement in biological process (BP) such as cell adhesion, T cell differentiation, and regulation of angiogenesis (Fig. 1E). These findings demonstrate that lung tissue from LPS-induced sepsis-ALI mice can be classified into seven distinct cell types that participate in BP including cell adhesion, T cell differentiation, and vascular development regulation.

### In the ALI, AMs aggregate into highly inflammatory and tissue-regenerative subgroups

**Fig. 2:**
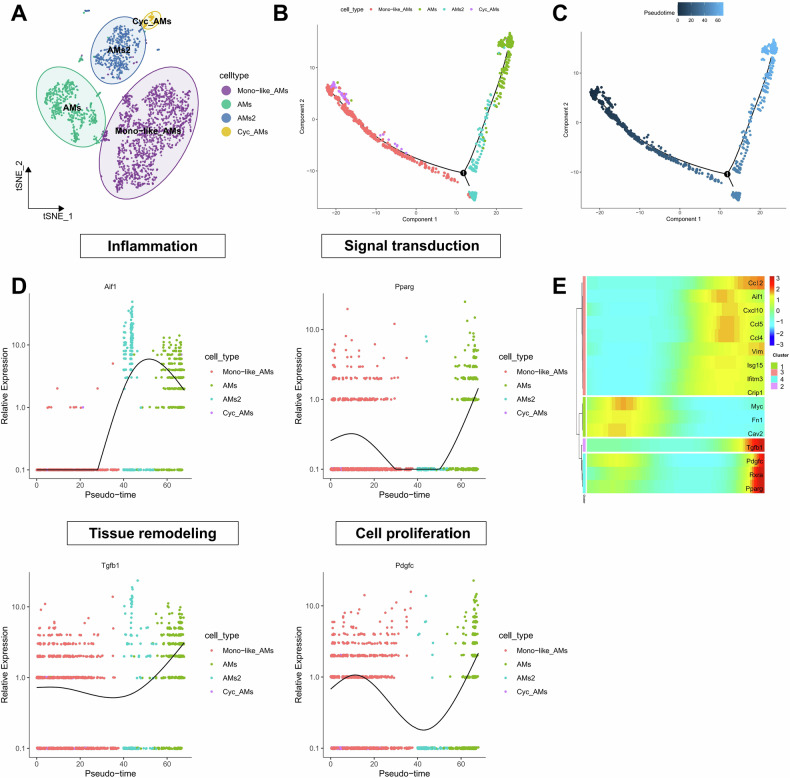
Analysis of AMs cell subtypes and pseudo-temporal analysis in LPS-induced Sepsis-ALI. **A** t-SNE distribution plot of AMs cell subtypes analysis; **B** Cluster distribution of AMs cell subtypes on the developmental tree. Different colors represent different cell subtypes; **C** Pseudo-temporal trajectory order of AMs cell subtypes arranged by pseudo-time values. Each point corresponds to a cell, where darker colors indicate lower pseudo-time values (distance from the root node); **D** Expression changes of Aif1 (inflammation), Pparg (alternative activation), Tgfb1 (tissue remodeling), and Pdgfc (cell growth) genes over pseudo-time; **E** Cluster heatmap displaying the expression of genes involved in antiviral host defense (Isg15, Ifitm3, Cxcl10), inflammation (Aif1), chemotaxis (Ccl2, Ccl4, Ccl5), as well as alternative activation (Pparg, Myc, Cav2, Rxra), tissue remodeling (Fn1, Vim, Tgfb1), and cell growth (Crip1, Pdgfc). The color gradient from blue to red indicates relative expression levels from low to high.

AMs are cells located in the alveolar interstitium that play a crucial role in maintaining tissue homeostasis and immediate host defense [15]. To map the composition and phenotype of AMs in the lungs of mice following LPS induction, we further extracted AM populations for subgroup analysis. The results showed that, based on gene expression types (Fig. S2A), cells were annotated using the online CellMarker website, and AMs were reclustered into four subgroups: Mono-like_AMs, Cyc_AMs, AMs, and AMs2 (Fig. 2A). To elucidate the transitional process of AM development, we conducted a pseudotime analysis on all AM subgroups. We found that Mono-like_AMs and Cyc_AMs were in the same state at the developmental starting point, indicating that macrophage subgroups evolve from Mono-like_AMs and Cyc_AMs and transition through AMs2 to AMs (Figs. 2B and S2B, C). Figure 2C depicts the proposed cellular timeline, with the highest cellular chronology value found in AMs. The gene expression patterns distributed along pseudotime indicate that Aif1 (inflammation) increases in AMs2 and decreases in AMs, while Pparg (signal transduction), Tgfb1 (tissue remodeling), and Pdgfc (cell growth) gene expression decreases in AMs2 and increases in AMs (Fig. 2D). Furthermore, we calculated the relevant differential genes associated with antiviral host defense (Isg15, Ifitm3, Cxcl10), inflammation (Aif1), chemotaxis (Ccl2, Ccl4, Ccl5), as well as those related to signal transduction (Pparg, Myc, Cav2, Rxra), tissue remodeling (Fn1, Vim, Tgfb1), and cell growth (Crip1, Pdgfc), and created a clustering heatmap showing that genes with similar expression patterns were grouped together. Genes related to alternative activation (Pparg, Rxra), tissue remodeling (Tgfb1), and cell growth (Pdgfc) were more highly expressed (Fig. 2E). Based on the above findings, we conclude that the AMs2 subgroup exhibits a pro-inflammatory phenotype, while AMs exhibit a transcriptional phenotype associated with tissue regeneration, similar to the TR-AM phenotype. These changes in gene expression patterns provide important insights into the process of tissue homeostasis restoration.

### The interaction between AMs and epithelial cells influences sepsis-induced ALI

**Fig. 3:**
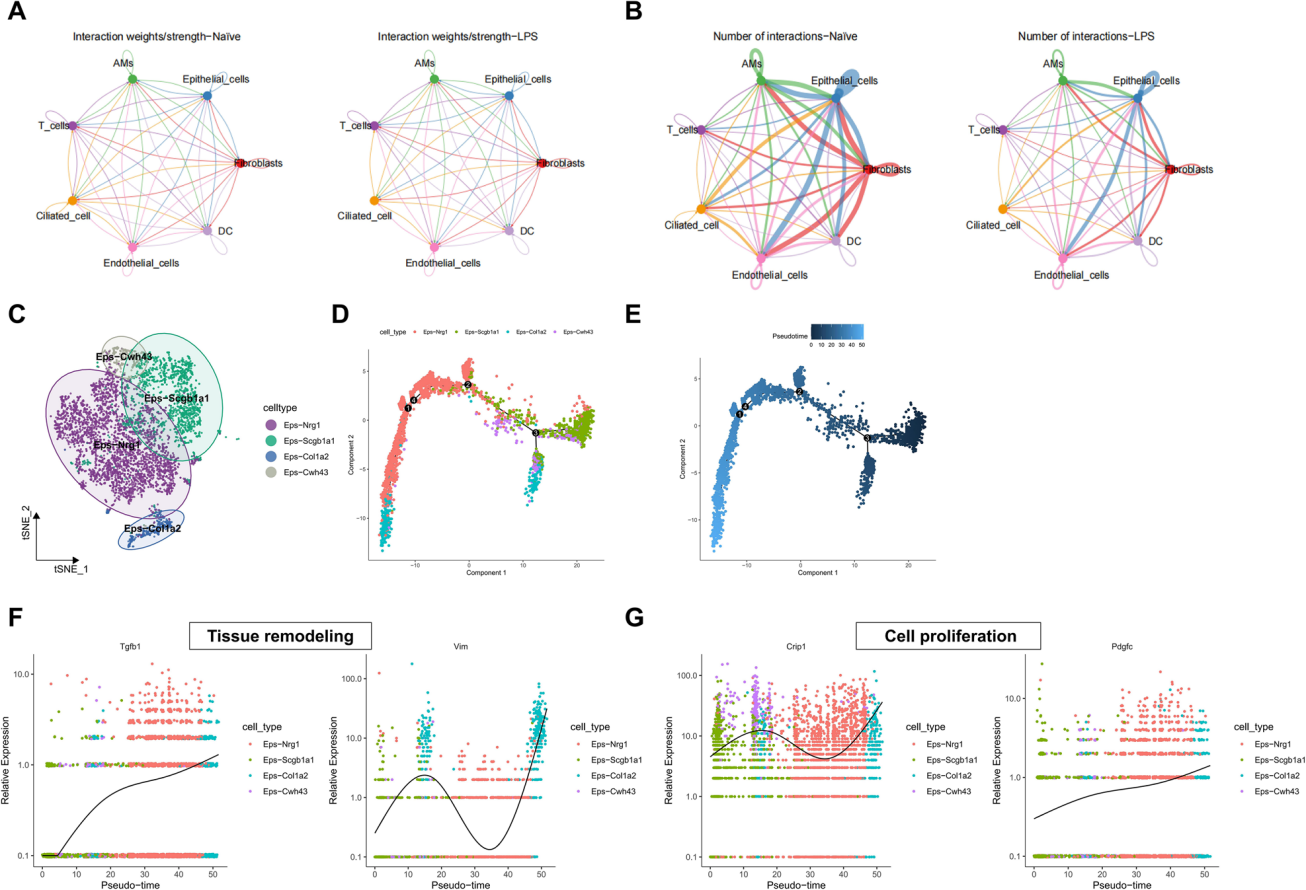
Interaction between AMs and epithelial cells in LPS-induced Sepsis-ALI and subcluster analysis of epithelial cells with pseudo-temporal analysis. **A** Cell communication circle plots in Naïve and LPS samples, where line thickness represents pathway interaction strength (**A**) and the number of pathways (**B**); **C** t-SNE distribution plot of epithelial cell subtypes analysis; **D** Cluster distribution of epithelial cell subtypes on the developmental tree. Different colors represent various cell subtypes; **E** Pseudo-temporal trajectory order of epithelial cell subtypes arranged by pseudo-time values. Darker colors indicate lower pseudo-time values (distance from the root node); **F**, **G** Expression changes of tissue remodeling genes (Vim, Tgfb1) and cell growth-related genes (Crip1, Pdgfc) over pseudo-time.

Previous studies have indicated that the coordinated action between macrophages and epithelial cells is considered essential for tissue repair [16, 17]. To further elucidate the mechanism of cellular evolution in LPS-induced Sepsis-ALI mouse models, we investigated the ligand-receptor-mediated intercellular communication within all cellular components. We simulated the expression of cell types in the immune microenvironment showing ligand-receptor interactions. The results revealed a strong interaction between epithelial cells and AMs. In the LPS group, the network diagram illustrated a decrease in interaction between AMs and epithelial cells (Fig. 3A, B). Furthermore, we explored the phenotypic changes of epithelial cells and performed subpopulation analysis based on gene expression profiles (Fig. S3A). The analysis revealed that epithelial cells were re-clustered into four subpopulations: Eps-Nrg1, Eps-Scgb1a1, Eps-Col1a2, and Eps-Cwh43 (Fig. 3C).

To elucidate the transitional process of epithelial cell development, we conducted pseudotime analysis on all epithelial cell subpopulations. We constructed a pseudotime developmental tree and identified four distinct branching points. Eps-Scgb1a1 was found to be situated at the developmental origin, Eps-Cwh43 predominantly located at branch point 3, Eps-Col1a2 positioned at the terminus of branch points 3 and 1, and Eps-Nrg1 exhibited the broadest distribution (Figs. 3D, E and S3B, C). The gene expression patterns along pseudotime distribution indicated differential gene expression of transcripts related to tissue remodeling (Vim, Tgfb1) and cell growth (Crip1, Pdgfc), showing an upward trend in Eps-Col1a2 and a downward trend in Eps-Nrg1 and Eps-Scgb1a1 subpopulations (Fig. 3F, G). Integrating the AMs subpopulations and the pseudo-temporal analysis results, we hypothesize that the interaction between transitional AMs and epithelial cells may promote epithelial repair in LPS-induced Sepsis-ALI towards the end of development, associated with tissue remodeling and cell growth.

### TR-AMs demonstrate proliferative and barrier-protective effects on AEC both in vivo and in vitro

**Fig. 4:**
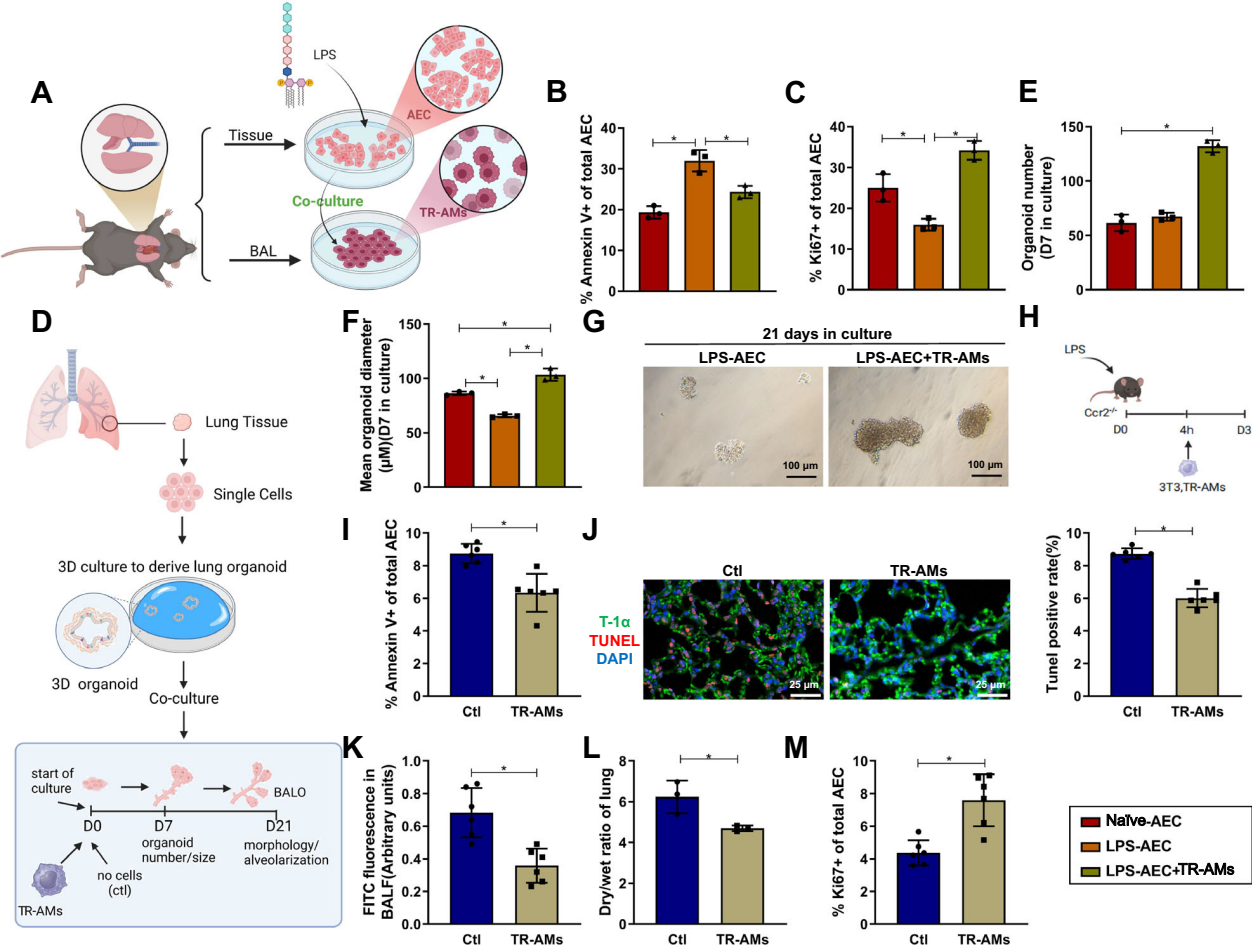
Role of TR-AMs in vitro and in vivo in AEC proliferation and barrier protection. **A** Schematic diagram of co-culture between AECs from control or LPS-induced mice and TR-AMs (Created with BioRender.com); **B**, **C** Percentages of apoptotic (Annexin V+, B) and proliferating (Ki67+, C) AECs after 24 h co-culture with TR-AMs; **D** Schematic diagram of BALO co-culture with TR-AMs (Created with BioRender.com); Quantification of organoid number (**E**) and diameter (**F**, µm) after 7 days of co-culture; **G** Representative images of organoids after 21 days of culture (EVOS FL microscope; scale bar: 100 µm). **H** Schematic of TR-AM transplantation into the lungs of Ccr2^−/−^ mice (Created with BioRender.com); **I** Flow cytometric analysis of AEC apoptosis percentage (3T3 group vs. TR-AMs group); **J** TUNEL and T-1α co-staining showing apoptosis of alveolar epithelial progenitor cells (green: TUNEL; red: T-1α; scale bar: 25 µm); **K** Quantification of FITC-albumin leakage (BALF/serum fluorescence ratio); **L** Lung wet/dry ratio; **M** Proliferation rate of AEC II (Ki67^+^). The in vitro cell experiments were repeated three times, with *n* = 6 for in vivo mice; * indicates *P* < 0.05.

To investigate the role of AMs on epithelial cells, as implied by transcriptomic analysis, we co-cultured flow cytometry-sorted TR-AMs (Fig. S4A) with LPS-induced mouse AEC in vitro (AEC, Fig. S4B) (Fig. 4A). Compared to the Naïve-AEC group, the LPS-AEC group exhibited increased apoptosis and decreased proliferation, while TR-AMs inhibited LPS-induced AEC apoptosis and promoted proliferation post-induction compared to LPS-AEC (Fig. 4B, C and S4C, D). The MTT assay revealed that the survival rate of cells in the LPS-AEC + TR-AMs group was not significantly different from that of TR-AMs alone, with the survival rate remaining above 90% (Fig. S4E). To validate the latter, we utilized the bronchioalveolar organoid (BALO) culture model, which features a proximal-distal pattern with branching airways and alveoli, allowing for simulation of lung development and epithelial cell differentiation (Fig. S4F, G), providing an overview of macrophage-epithelial cell interactions (Fig. 4D). TR-AMs supported the generation of BALOs (in terms of quantity and size), indicating that the presence of TR-AMs promoted epithelial cell proliferation and organoid differentiation (Fig. 4E–G). To confirm the robustness of the TR-AMs phenotype beyond their local (anti-inflammatory) microenvironment, we developed a short-term induction model and applied in vivo-generated TR-AMs to LPS-induced Ccr2^-/-^ mice via pulmonary transfer (Fig. 4H). Compared to the Control group (no cells), the damaging effect of TR-AMs was substantially lower (quantified by AEC apoptosis; Fig. 4I). TUNEL and immunofluorescence analyses showed a significant reduction in the apoptosis rate of alveolar epithelial progenitor cells in mouse lung tissue treated with TR-AMs compared to the Control group (no cells) (Fig. 4J). TR-AMs induced the proliferation of CD45/31negEpCamlowT1-αneg alveolar epithelial progenitor cells (AEC II), which improved barrier function on day 7 post-induction, reducing the lung wet/dry ratio in mice (Fig. 4K–M). Taken together, these data confirm the regenerative function of TR-AMs on the alveolar epithelium, validating the functional heterogeneity of macrophage subpopulations identified through scRNA-seq in both in vitro and in vivo models.

### The role of Sbno2 in TR-AMs drives AEC regeneration and promotes alveolar epithelial barrier function

To further investigate the regulatory mechanism of TR-AMs on lung epithelial cells, we isolated TR-AMs from the BALF of four naive and LPS-treated mice for transcriptome sequencing analysis. Using |log2 (Foldchange)| > 1 and a significance of *P* < 0.05 as the threshold, we identified 289 differentially expressed genes (Tables S1-2), comprising 192 upregulated and 97 downregulated genes (Fig. S5A). Subsequently, integrating the differential marker genes of AMs and epithelial cells from naive and LPS single-cell transcriptome sequencing, we obtained 692 and 423 differentially expressed genes, respectively, with |log2(Foldchange)| > 0.25 and significant *P* < 0.05 as thresholds (Fig. S5B, C). Among the intersection of these gene sets, three candidate genes were identified: Sgms2, Sfn, and Sbno2 (Fig. S5D). Through bubble plots and violin plots, we illustrated the expression distribution of these three genes in various cell populations in the single-cell transcriptome, showing that both Sgms2 and Sbno2 were abundantly expressed in AMs and epithelial cells (Fig. S5E, F). Furthermore, we demonstrated the expression of Sgms2, Sfn, and Sbno2 in subpopulations of AMs, revealing that only Sbno2 was highly enriched in TR-AMs exhibiting a distinct phenotype, and its expression in LPS-treated AMs was slightly elevated compared to naive AMs. Temporal expression analysis indicated fluctuations of the three genes in AM subpopulations and epithelial cells, with Sbno2 especially upregulated in the TR-AM subpopulation displaying a distinctive phenotype and in the Eps-Col1a2 subpopulation associated with tissue remodeling and cell growth (Fig. S6A–D).

**Fig. 5:**
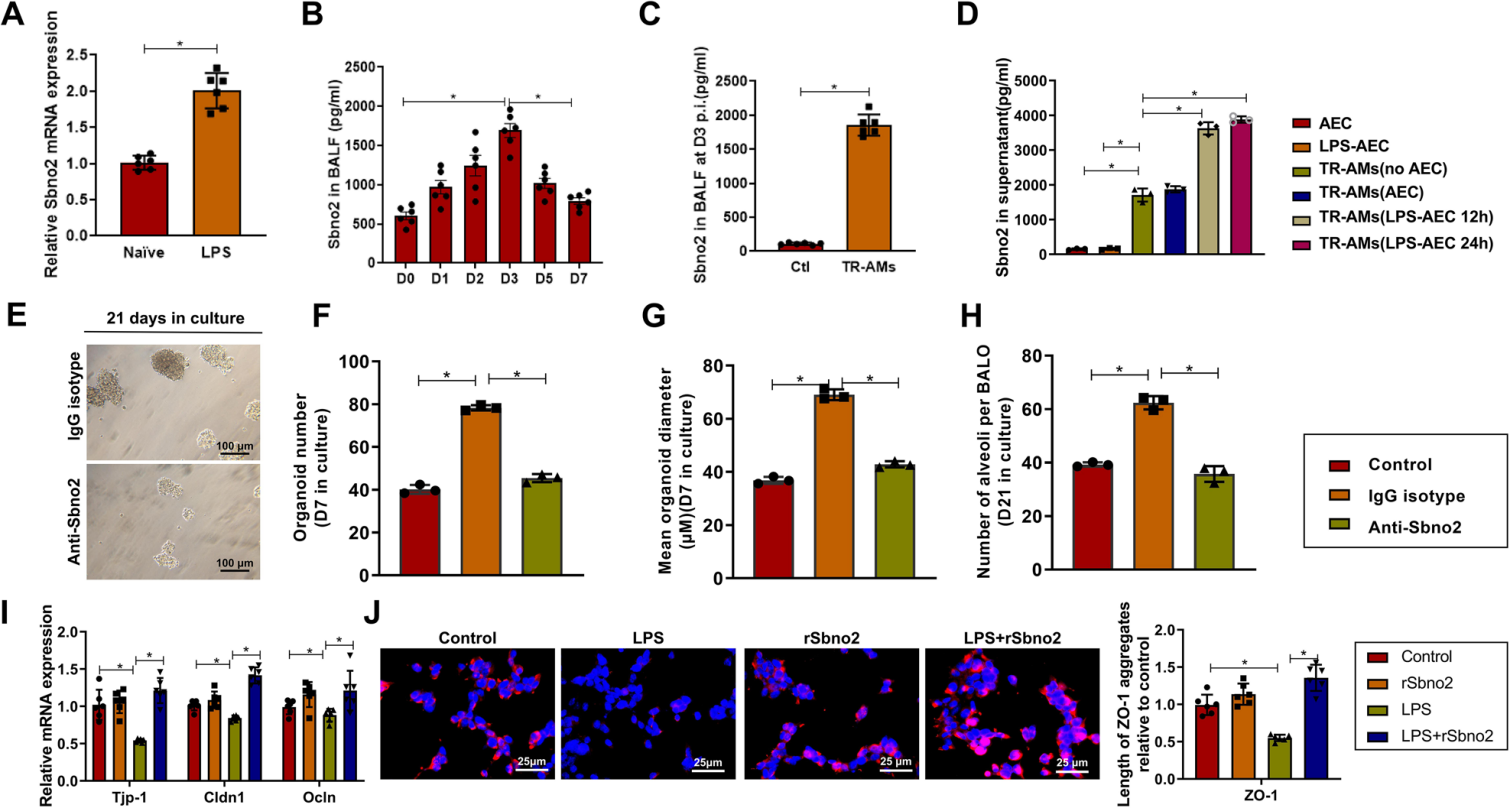
Impact of Sbno2-expressing TR-AMs on AEC expansion and alveolar epithelial barrier function in LPS-induced Sepsis-ALI. **A** Comparative analysis of Sbno2 mRNA expression in TR-AMs from mice 21 days post LPS induction by qPCR; **B** Quantification of soluble Sbno2 in BALF at days 0/1/2/3/5/7 post-LPS induction in mice; **C** Quantification of soluble Sbno2 in BALF using TR-AMs subpopulations transplanted into BALF from LPS-induced Ccr2^−/^^−^ mice given TR-AMs subpopulations isolated from BALF of LPS-induced wild-type mice at specified time points; **D** ELISA detection of Sbno2 concentration in supernatants of TR-AMs isolated from culture medium of non-LPS-induced or induced AEC (24 h) and BALF treated with TR-AMs for 12 h in medium from non-LPS-induced or induced AECs or untreated TR-AMs; **E** Representative images of BALOs co-cultured with TR-AMs for 21 days and treated with anti-Sbno2 mAb or isotype control. The scale bar represents 100 µm; **F** Quantification of BALO numbers, with individual data points representing the mean organ count per well; **G** Average diameter (µm) of organoids per well after 7 days, with individual data points representing the average diameter of organoids per well; **H** Average number of alveoli in BALOs after 21 days (control) or co-cultured with AMs and anti-Sbno2 mAb or isotype control; **I** Quantification of tight junction gene expression (Tjp-1, Cldn1, Ocln) in LPS-induced mouse AECs treated with rSbno2 (20 ng/mL) via qPCR; **J** Representative images of ZO-1 localization in LPS-induced and/or rSbno2-treated AEC monolayers. The scale bar represents 25 µm. In vitro cell experiments were repeated three times, with *n* = 6 for in vivo mice; * indicates *P* < 0.05.

Prior research has suggested that Sbno2 in macrophages functions as a component of the IL-10 regulatory pathway, inhibiting the expression of inflammatory genes [14]. Here, we delved into whether Sbno2 affects the functionality of TR-AMs. Initially, qRT-PCR confirmed the expression of Sbno2 in TR-AMs induced by naive and LPS treatments, showing a significant increase in Sbno2 expression in LPS-induced TR-AMs compared to naive TR-AMs (Fig. 5A). ELISA analysis of Sbno2 levels in BALF from LPS-induced mice over different time intervals demonstrated a substantial increase starting from the injury phase, peaking at day 3, sustaining through the resolution phase till day 5, and declining by day 7 (Fig. 5B). Subsequently, on day 7 post-induction following cell transplantation of TR-AMs, we quantified Sbno2 in Ccr2^−/−^ mice. We observed that TR-AMs promoted the expression of soluble Sbno2 in BALF (Fig. 5C), suggesting that inflammation or lung alveolar microenvironmental injury induces Sbno2 expression or release by TR-AMs. To investigate whether soluble mediators released from injured AECs during the induction process are involved, TR-AMs were stimulated with conditioned media from LPS-induced and non-induced AECs. Interestingly, while Sbno2 was undetectable in the supernatant without TR-AMs, significantly increased levels of Sbno2 released by TR-AMs were observed in conditioned media from AECs induced for 12 or 24 h, signifying an upregulation of Sbno2 expression upon LPS-induced AEC signaling (Fig. 5D). These results indicate that TR-AMs may mediate alveolar epithelial regeneration through Sbno2 expression.

To validate our hypothesis that Sbno2-expressing TR-AMs support AEC growth, we co-cultured BALOs with AMs under conditions with or without anti-Sbno2 monoclonal antibody (mAb) or isotype control (IgG) antibodies. In the presence of AMs expressing high levels of Sbno2 (IgG isotype), the number and size of organoids significantly increased, a response that was counteracted by anti-Sbno2 treatment by the 7th day of culture, leading to a reduction in the number of fully developed alveoli in the BALOs (Fig. 5E–H). Furthermore, considering the beneficial effects of TR-AMs on alveolar barrier function in vivo (Fig. 4J), we investigated the potential mechanisms by which rSbno2 mediates AEC barrier function improvement, analyzing the expression of tight junction genes in the context of LPS-induced injury. In vitro, cultures of mouse AECs induced with LPS showed decreased mRNA expression of Tjp-1 (encoding ZO-1, a tight junction protein 1), with lesser reductions in tight junction genes Cldn1 or Ocln (encoding claudin-1 and occludin). Treatment with rSbno2, compared to the LPS group, increased the expression of these three genes (Fig. 5I) and raised the levels of ZO-1 protein as detected by immunofluorescence (Fig. 5J). These findings underscore that Sbno2 in TR-AMs supports AEC growth, enhancing the alveolar epithelial barrier function.

### Knockout of Sbno2 in TR-AMs inhibits alveolar epithelial progenitor cell (AEC II) proliferation and lung barrier function

**Fig. 6:**
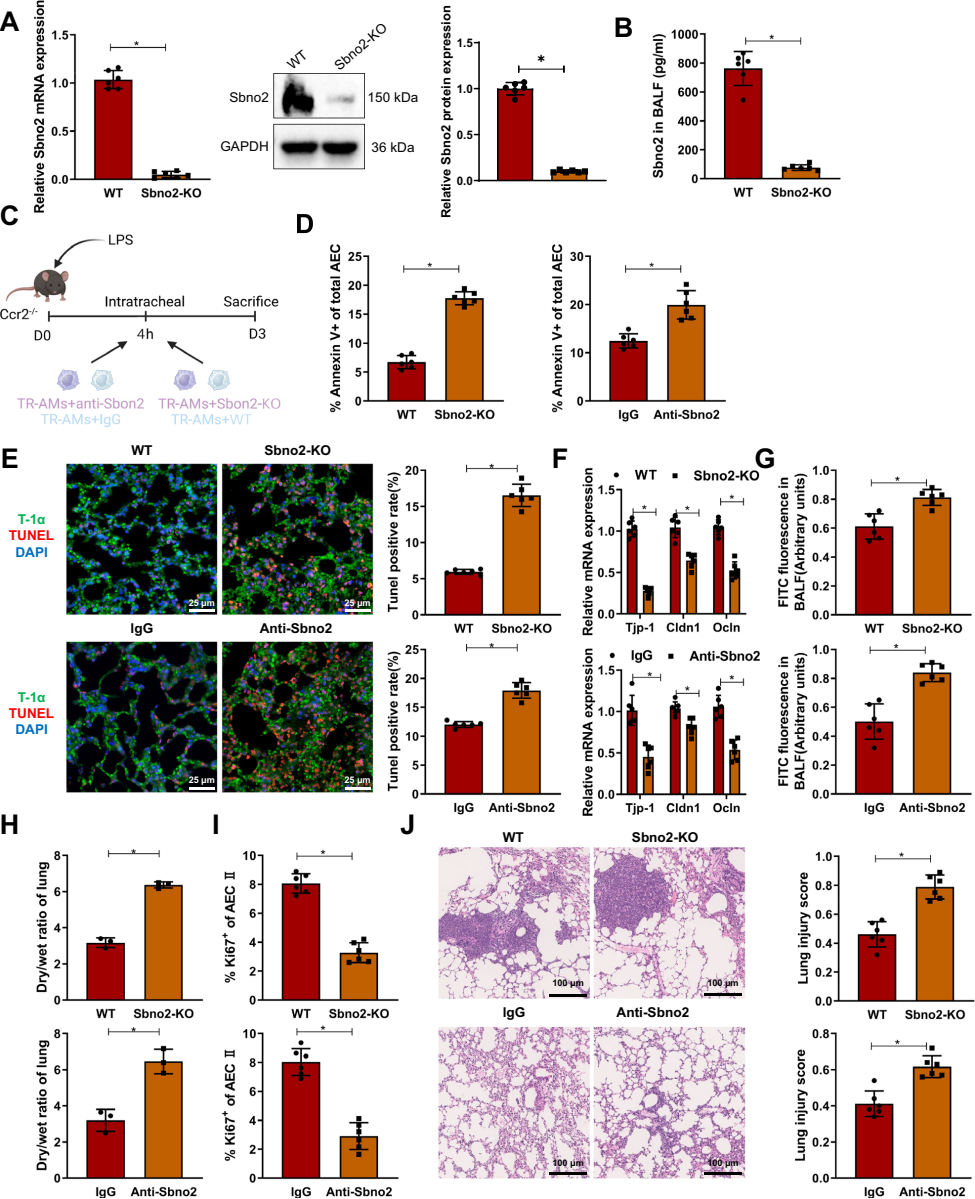
Impact of Sbno2 knockout on lung epithelial regeneration and lung barrier function. **A** qRT-PCR and Western Blot analysis of Sbno2 mRNA and protein expression in TR-AMs sorted from BALF of control WT mice and Sbno2-KO mice; **B** ELISA measurement of Sbno2 content in BALF of WT and Sbno2-KO mice; **C** Experimental schematic analyses from TR-AMs of Sbno2-KO mice or TR-AMs pre-incubated with anti-Sbno2 antibody to establish the TR-AMs→Ccr2^−/−^ mouse lung model (Created with BioRender.com); **D** Percentage of Annexin V+ AECs; **E** Apoptosis rate of alveolar epithelial progenitor cells in mouse lung tissue detected by Tunel and immunofluorescence (T-1α serves as an AEC-specific marker, expressed in the cytoplasm, while Tunel-positive cells are expressed in the nucleus); **F** qRT-PCR measurement of lung tight junction protein mRNA expression levels; **G** Quantitative analysis of barrier function disruption in BALF through FITC-albumin fluorescence assay; **H** Lung wet/dry weight ratio; **I** Percentage of Ki67^+^ proliferating AEC II; **J** Representative lung tissue section images (H&E staining) and lung injury scores. The scale bar represents 100 µm. *n* = 6 for in vivo mice; * indicates *P* < 0.05.

To ascertain the pivotal role of Sbno2 as a key mediator in vivo for TR-AMs-mediated epithelial cell protection, we established a TR-AMs→Ccr2^−/−^ mouse lung model utilizing TR-AMs derived from Sbno2-KO mice or pre-incubated with anti-Sbno2 monoclonal antibodies, analyzing AEC parameters at the peak of lung injury on day 3. By crossing with cre-deficient mice to generate Sbno2-KO mice, the absence of Sbno2 mRNA, protein and tdTomato reporter gene in LPS-induced Sbno2-KO TR-AMs compared to cre-negative WT mice confirmed the Sbno2-KO status (Fig. 6A–C). The specificity of anti-Sbno2 antibodies has been confirmed through Western blot analysis, displaying a single band at the expected molecular weight in WT TR-AMs, while absent in Sbno2-KO samples (Fig. 6A). Compared to the control group, the antibody or knockout of Sbno2 in TR-AMs reduced the beneficial effects of TR-AMs, leading to increased AEC apoptosis, decreased expression of tight junction genes in epithelial cells, impaired alveolar barrier function, increased lung wet/dry ratio, and reduced proliferation of AEC II (Fig. 6D–I). Finally, Ccr2^−/−^ mice treated with recombinant Sbno2 or anti-Sbno2 antibodies showed sustained tissue inflammation on day 7 compared to the control group (Fig. 6J).

**Fig. 7:**
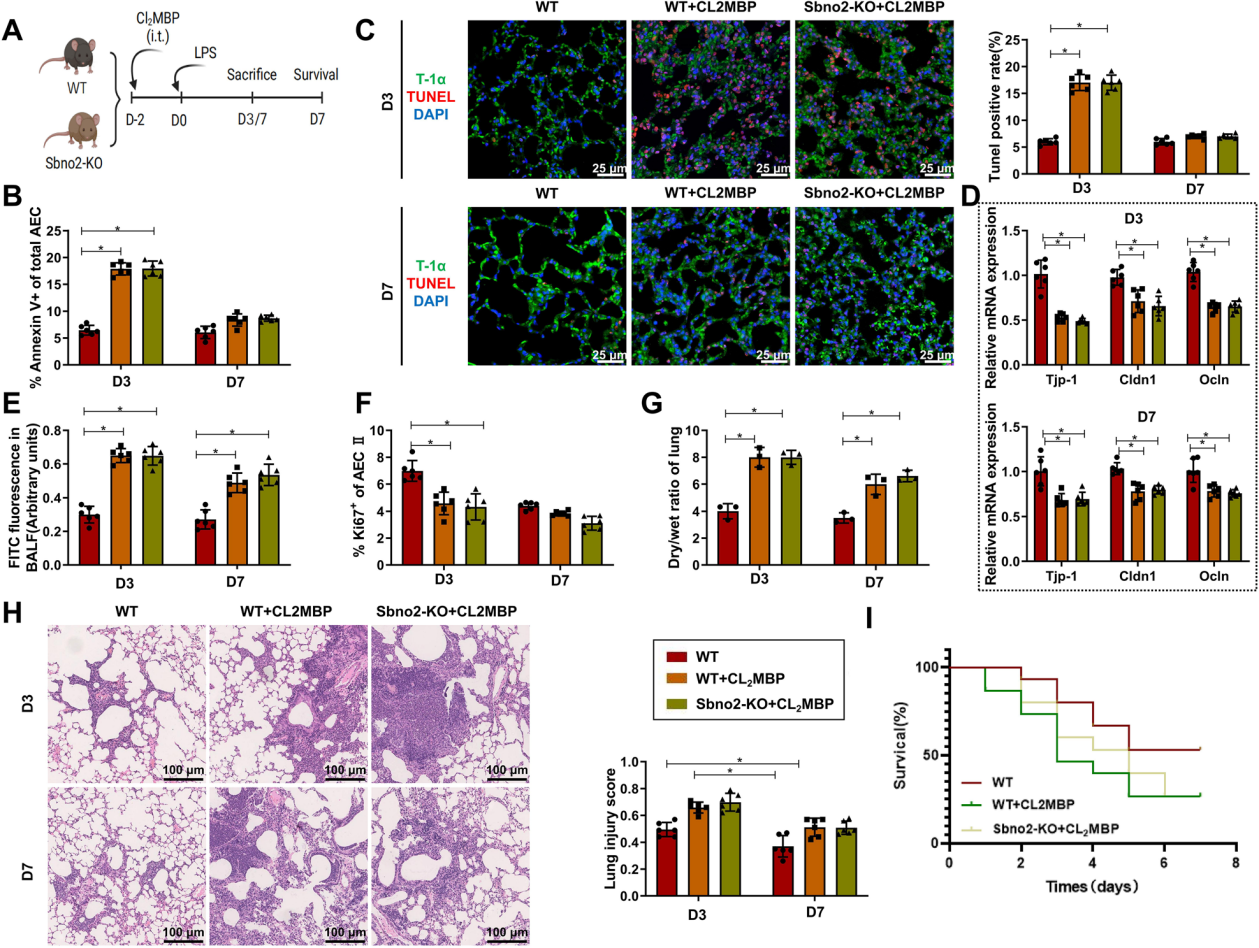
Deletion of Sbno2 in TR-AMs affects AEC II expansion and lung barrier function in LPS-induced Sepsis-ALI mice. **A** Experimental workflow of intranasal CL_2_MBP injection in WT or Sbno2-KO mice (Created with BioRender.com); **B** Percentage of Annexin V+ AECs at days 3 and 7 in Sbno2-KO and WT mice with or without CL_2_MBP treatment; **C** TUNEL and immunofluorescence detection of alveolar epithelial progenitor cell apoptosis in mouse lung tissue (T-1α is a specific marker for AECs, expressed in the cytoplasm, with TUNEL-positive expression in the nucleus). **D** mRNA expression levels of tight junction component genes in lung tissue (qPCR) in Sbno2-KO and WT mice with or without CL2MBP treatment on days 3 and 7; **E** Quantitative analysis of FITC-albumin fluorescence in BALF at days 3 and 7 in Sbno2-KO and WT mice with or without CL_2_MBP treatment to assess barrier function disruption; **F** Percentage of Ki67^+^ AEC II at days 3 and 7 in Sbno2-KO and WT mice with or without CL2MBP treatment; **G** Lung wet/dry ratio in mice; **H** Lung histopathological analysis and lung injury quantification of WT or Sbno2-KO mice with or without CL_2_MBP treatment obtained at D3/7 p.i. (H&E staining). The scale bar represents 100 µm; **I** Survival analysis of mice induced by high doses of LPS, Survival analysis of Sbno2-KO mice with or without CL_2_MBP treatment or WT mice. Except for *n* = 15 in survival analysis, *n* = 6 for other mice per group. * indicates *P* < 0.05.

To further explore the role of TR-AMs in lung epithelial cells, we intranasally administered CL_2_MBP to WT or Sbno2-KO mice (Fig. 7A) to suppress TR-AMs (Fig. S7A). Next, we analyzed parameters of sustained alveolar injury in Sbno2-KO and WT mice with or without CL_2_MBP treatment, starting around day 3 after LPS induction. On day 3 post-induction, WT + CL_2_MBP mice showed significantly increased AEC apoptosis, reduced AEC II proliferation, and decreased expression of tight junction genes (Tjp-1, Cldn1, Ocln) compared to WT mice. Elevated alveolar barrier leakage persisted until day 7 post-induction, with an increased lung wet/dry ratio in mice (Fig. 7B–G). Histological examination of lung tissues showed progressive lung injury in WT + CL_2_MBP mice compared to WT mice (Fig. 7H). Furthermore, post-induction survival rates from day 3 to day 7 were significantly lower in Sbno2-KO mice compared to WT mice when induced with virus doses causing low mortality in control mice (Fig. 7I). Interestingly, there was no significant difference observed between WT + CL_2_MBP and Sbno2-KO + CL_2_MBP mice in terms of AEC II proliferation and lung barrier function. Sbno2 was undetectable in the BALF of WT and Sbno2-KO mice treated with CL_2_MBP on days 3 and 7 post-induction compared to WT control mice, indicating that TR-AMs are the primary source of Sbno2 in the alveolar interstitium (Fig. S7B, C). In addition, by comparing the phenotypes of WT and Sbno2-KO mice, we found that under basal conditions, Sbno2-KO mice did not exhibit spontaneous pulmonary abnormalities or significant changes in the number of AMs (Fig. S7A), and no notable alterations were observed in Sbno2-expressing cells such as AMs. However, following LPS stimulation, Sbno2-KO mice displayed markedly impaired alveolar epithelial regeneration and anti-inflammatory responses, evidenced by increased AEC apoptosis (Figs. 6D and 7B), downregulation of tight junction gene expression (Figs. 6F and 7D), and elevated lung wet/dry ratio (Figs. 6H and 7F). These results suggest that Sbno2 plays a critical regulatory role in TR-AM-mediated regeneration and anti-inflammatory function under stress conditions, rather than in maintaining homeostasis under baseline conditions.

These findings underscore that knockout of Sbno2 in TR-AMs inhibits alveolar epithelial progenitor cell (AEC II) proliferation and lung barrier function.

### Therapeutic Efficacy of Intratracheally Administered rSbno2 in LPS-Induced Sepsis-ALI Mice

**Fig. 8:**
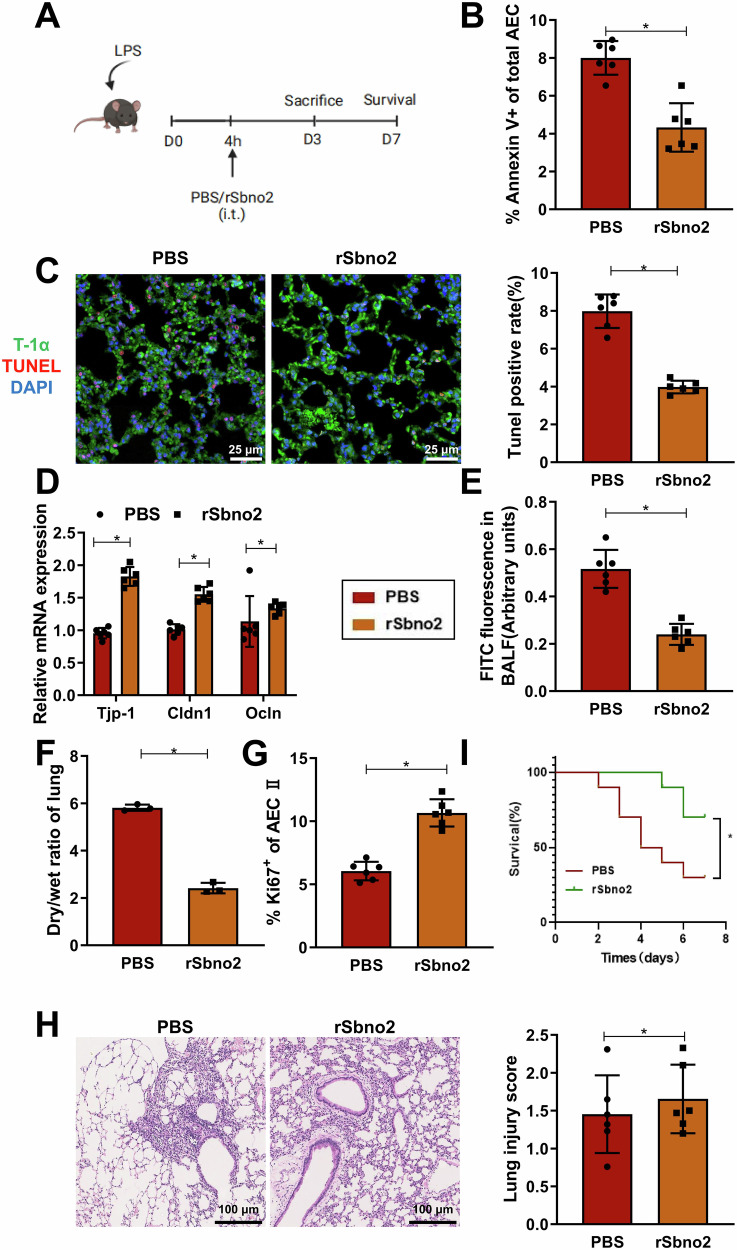
Protective effect of rSbno2 treatment on lung epithelial barrier function in lethally LPS-induced and rescued mice. **A** Schematic diagram of tracheal administration of rSbno2 (5 µg) or PBS on 4 h post-LPS induction (Created with BioRender.com); **B** Percentage of Annexin V^+^ AECs; **C** TUNEL and immunofluorescence assays to detect apoptosis of alveolar epithelial progenitor cells in mouse lung tissue (T-1α as an AEC-specific marker, expressed in the cytoplasm, with TUNEL positivity in the nucleus); **D** qRT-PCR analysis of tight junction component gene mRNA expression in flow-sorted EpCam+ cells; **E** Quantification of barrier function disruption through FITC-albumin fluorescence analysis; **F** Lung wet/dry ratio in mice; **G** Percentage of Ki67^+^ proliferating AEC II; **H** Lung tissue sections of mice on day 3 post-LPS induction stained with H&E. The scale bar represents 100 µm; **I** Survival analysis of mice induced by high doses of LPS, Survival rates of LPS-induced mice treated with PBS or rSbno2. Except for *n* = 10 in survival analysis, and *n* = 6 for other mice per group; * indicates *P* < 0.05.

To further investigate the therapeutic effects of Sbno2 in Sepsis-ALI mice, we assessed the potential of tracheal administration of rSbno2 on the 3rd day post-induction. The experimental setup (Fig. 8A) aimed to alleviate peak lung injury observed on the 3th day post-induction in a severe LPS-induced model. Treatment with rSbno2 reduced tissue inflammation associated with injury, decreased inflammatory cell infiltration and hemorrhage in the vascular wall or alveolar cavity, reduced apoptosis of AECs, upregulated the expression of tight junction genes Tjp-1, Ocln, and Cldn1, improved alveolar barrier function, and induced significant proliferation of AEC II cells, indicating a tissue-protective and regenerative effect (Fig. 8B–H). Finally, in mice induced with a lethal dose of LPS, treatment with rSbno2 demonstrated a significantly enhanced therapeutic effect compared to PBS control treatment (Fig. 8I), suggesting that local administration of rSbno2 may serve as a potential therapeutic strategy for Sepsis-ALI.

## Discussion

The findings of this study hold significant implications for the understanding and treatment of ALI. The regeneration of AECs is crucial for repairing damaged lung tissue, with TR-AMs playing a highlighted role in immune regulation throughout this process [7, 18, 19]. Sbno2, as a novel negative feedback regulator of IL-6, affects the antibacterial activity of macrophages and is involved in the inflammatory response [12, 13]. By delving into the mechanistic role of Sbno2 in TR-AMs, we can not only enhance our understanding of the functionality of pulmonary immune cells but also potentially offer new directions and foundations for therapeutic strategies for ALI.

The application of scRNA-seq technology has provided a more precise and comprehensive cellular-level analysis in this study. The high expression of Sbno2 in TR-AMs reveals a significant advantage in understanding the critical role these cells play in lung injury [7, 20, 21]. This innovative approach not only broadens our comprehension of interactions among alveolar cells but also deepens our understanding of the pathogenesis of lung diseases, providing a more thorough perspective on research related to lung injury.

This study, through scRNA-seq and functional experiments, reveals that TR-AMs expressing Sbno2 play a critical role in sepsis-induced ALI by promoting alveolar epithelial regeneration and barrier restoration. Database searches using the Human Protein Atlas showed high Sbno2 expression in human AMs, consistent with expression patterns observed in the mouse model, further indicating the translational potential of Sbno2 in lung injury repair.

Previous studies have shown that Sbno2 is induced by IL-10 and participates in NF-κB inhibition. Specifically, IL-10 induces the expression of the DExD/H helicase family co-repressor Strawberry notch homolog 2 (Sbno2) in a STAT3-dependent manner. Sbno2 inhibits NF-κB but not IRF7, suggesting its role as a negative regulator of pro-inflammatory cascades [22]. Additionally, IL-10 induces the expression of ETS family transcriptional repressor ETV3 and Sbno2 in both mouse and human macrophages, and this induction depends on STAT3 activation and TLR pathway stimulation. ETV3 and Sbno2 contribute to the anti-inflammatory effects downstream of IL-10 [14].

Mechanistically, we provide direct experimental evidence that TR-AMs can secrete Sbno2 into the alveolar space, and that conditioned medium from LPS-treated AECs significantly enhances this secretion. Although we have not yet visualized Sbno2 uptake by AECs, exogenous rSbno2 significantly promotes AEC proliferation and upregulates tight junction genes (Tjp-1, Cldn1, Ocln), while anti-Sbno2 antibody treatment inhibits BALO formation and alveolar differentiation. These findings strongly suggest a direct regulatory role of extracellular Sbno2 in epithelial regeneration. However, we cannot entirely exclude indirect mechanisms involving other paracrine factors or cell-cell interactions, and further studies using conditional genetic models or co-culture systems are needed to clarify the regulatory network.

Transcriptome analysis of TR-AMs from LPS-treated and control mice revealed that differentially expressed genes were enriched in regeneration-related pathways (e.g., proliferation, tight junction formation) and inflammatory networks (e.g., IL-10/ETV3/Sbno2 pathway). These genes were significantly downregulated upon Sbno2 knockout, consistent with previous reports on IL-10-mediated anti-inflammatory regulation in macrophages [14]. Together, these results support the core role of Sbno2 in TR-AM-mediated alveolar repair.

This study comprehensively demonstrates the essential function of Sbno2 in regulating inflammation and promoting AEC proliferation and differentiation via TR-AMs, establishing the Sbno2–TR-AM axis as a key regulatory pathway in alveolar regeneration. In ALI, Sbno2 is upregulated in TR-AMs and exerts protective effects on AECs, significantly improving epithelial barrier function and reducing tissue damage. Comparative in vitro and in vivo models further indicate that Sbno2 modulates TR-AM function under stress conditions, influencing ALI progression and prognosis. These findings enhance our understanding of local immune–epithelial interactions and provide theoretical and experimental foundations for targeted treatment of sepsis-induced ALI.

Despite these advances, several limitations must be acknowledged before translating these findings into clinical application. First, current results require validation in larger, more rigorous clinical studies to ensure reproducibility and generalizability. Based on previous findings that Sbno2 plays an important role in Sepsis-ALI progression, we evaluated the therapeutic potential of intratracheally delivered rSbno2 on day 3 post-LPS induction and observed significant tissue protection. However, we did not assess the effects of rSbno2 under non-LPS conditions, nor did we investigate its pharmacokinetics, half-life, or biodistribution—all of which are necessary for clinical translation. The long-term safety profile of rSbno2 (e.g., risk of fibrosis or chronic toxicity) remains to be evaluated, and factors such as inter-individual variability, drug safety, and efficacy assessments also warrant systematic investigation.

Furthermore, this study did not include phenotypic rescue experiments using exogenous rSbno2 in Sbno2-KO mice, which would be critical to clarify therapeutic potential. Although we validated changes in tight junction genes (Tjp1, Cldn1, Ocln) and the proliferation marker Ki67 in AECs treated with rSbno2 using qPCR and flow cytometry, we have not yet performed RNA-seq on rSbno2-treated AECs. Future studies will incorporate multi-omics approaches to comprehensively decode the molecular regulatory network. While we detected soluble Sbno2 in BALF and confirmed its functional effects upon exogenous addition, its uptake pathway and downstream signaling (e.g., NF-κB, STAT3) in AECs remain unclear. Future work will employ live-cell imaging, endocytosis inhibitors, and pathway-specific blockers to explore the mechanisms of action.

It is also worth noting that the recombinant Sbno2 protein used in this study was mouse isoform 1 (MyBioSource MBS1370624). Previous studies suggest that different Sbno2 isoforms may have functional heterogeneity in macrophage antibacterial activity [13], warranting future comparisons of isoform-specific effects on alveolar repair. Although all antibodies used in this study were validated by isotype controls, the absence of independent biochemical validation (e.g., Western blot, mass spectrometry) may affect the rigor of molecular conclusions. We will address this in follow-up experiments. Additionally, while Sbno2 is traditionally considered a nuclear transcriptional co-regulator, our findings indicate extracellular functional activity, suggesting that it may be released via non-classical pathways such as exosomes—consistent with our detection of soluble Sbno2 in BALF. The exact secretion mechanism requires further investigation.

The significance of this study lies in its first systematic identification of Sbno2 as a non-classically secreted immunoregulatory molecule, challenging the traditional view of it solely as a nuclear factor. We propose a novel model of “immune cell–repair factor–epithelial cell” cooperation, which provides new insights into TR-AM-based regenerative therapies for the lung and lays the foundation for personalized, cell-targeted interventions for ALI.

Although we have made substantial progress in elucidating this mechanism, several important scientific questions remain unresolved. Future studies will focus on three directions: (1) systematic evaluation of rSbno2 pharmacokinetics, safety, and off-target risks in different pathological contexts to support clinical translation; (2) phenotypic rescue experiments in Sbno2-KO models and comparison of isoform-specific functions to determine the optimal therapeutic target; and (3) application of integrated multi-omics analyses (e.g., scRNA-seq, proteomics, phosphoproteomics) and live imaging to dissect the dynamic signaling interactions between TR-AMs and AECs.

**Fig. 9:**
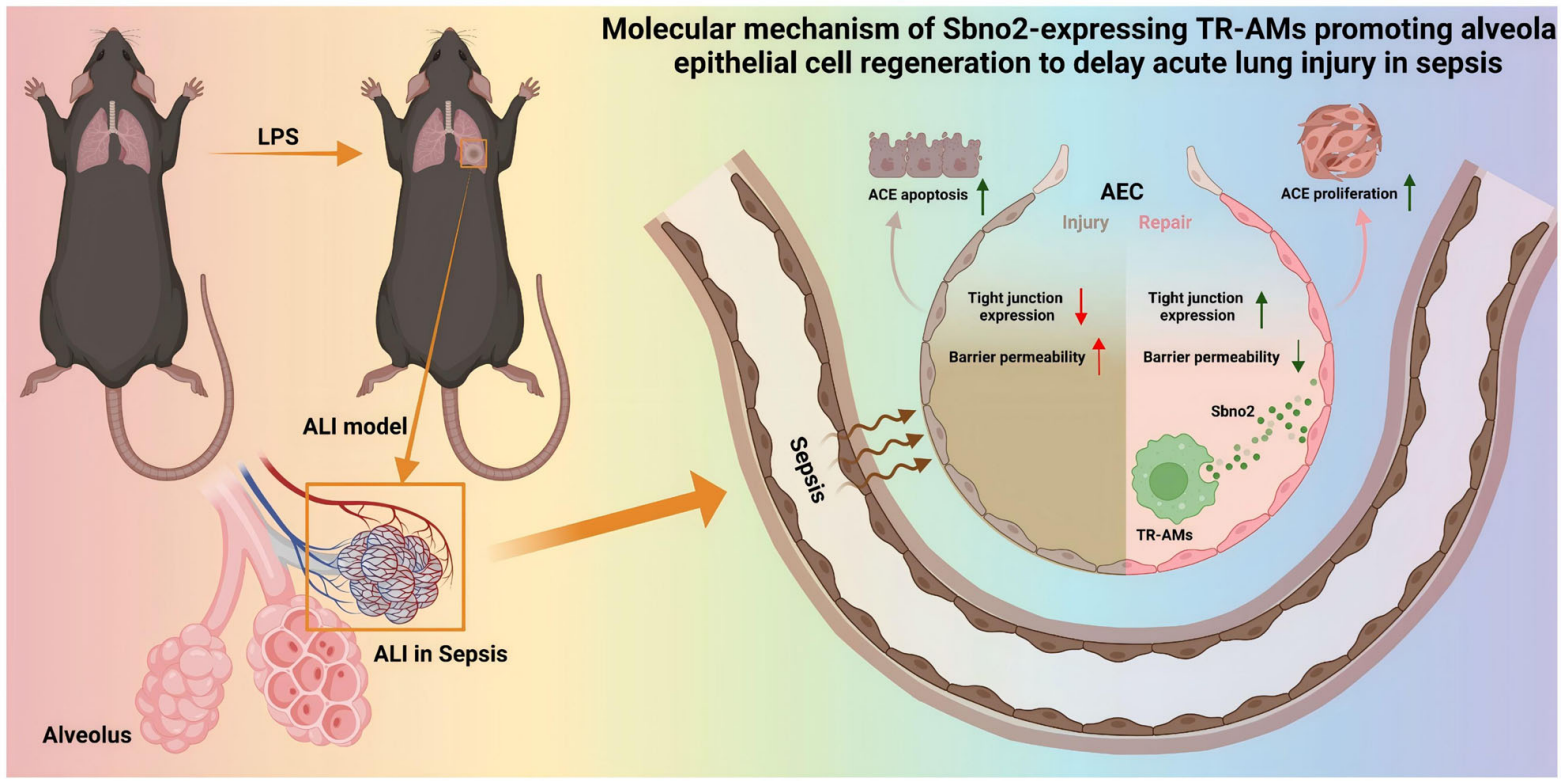
Schematic illustration of the molecular mechanisms by which Sbno2-expressing TR-AMs promote AEC regeneration and delay sepsis-induced ALI (Created with BioRender.com).

In conclusion, this study expands our understanding of the pathophysiology of sepsis-induced ALI, highlights the central role of alveolar immune cells in tissue regeneration, and identifies Sbno2 as a promising therapeutic target (Fig. 9). These findings not only advance research on pulmonary immune-regeneration interactions but also offer a new avenue for precision therapy in patients with ALI.

## Materials and methods

### Experimental animals

All mouse experiments conducted in this study strictly adhered to international and domestic ethical guidelines and regulations regarding the protection of experimental animals. The experimental procedures were approved by the institutional ethical review committee and carried out in compliance with its regulations. All mice were individually housed in ventilated cages with controlled temperature and humidity (22.5 °C, 52.5%), maintained on a 12:12 h light–dark cycle, and provided with standard diet and ad libitum water. Male mice aged 8–20 weeks (20–30 g) were selected for the experiments. Wild-type C57BL/6 (NTG, Vital River Laboratory Animal Technology Co., Ltd, Beijing, China), C57BL/6 background Sbno2-KO (S-KO-05142), and Ccr2-KO (Ccr2^−/−^, S-KO-01543) mice were purchased from Saiye (Suzhou) Biotechnology Co., Ltd.

An LPS-induced ALI model was established in 8-week-old female C57BL/6 mice devoid of pathogens. Briefly, mice were anesthetized with isoflurane, and following skin and muscle incisions, the trachea was exposed. A slow injection of 100 μL of 1 mg/kg LPS (Escherichia coli serotype 055:B5, L2880, Sigma-Aldrich, USA) was administered using a micro syringe from the distal end of the trachea. The presence of crackles in the lungs indicated a successful model, after which the incision was sutured. Mice were euthanized with pentobarbital via intraperitoneal injection at different time points (0–7 d), and lung tissue and bronchoalveolar lavage fluid (BALF) were collected for analysis. BALF was collected by instilling 1 mL of PBS into the trachea, repeating the lavage three times. For histological analysis, lung tissue sections were fixed in 4% paraformaldehyde (PFA), embedded in paraffin, and stained with H&E. Ten to fifteen random fields from 5 μm sections at 200× magnification were selected using a Leica microscope (Leica, Wetzlar, Germany). The severity of ALI was assessed using the McGuigan scoring system based on alveolar wall thickening or hyaline membrane formation, alveolar congestion, inflammatory cell infiltration in vessel walls or alveolar spaces, and hemorrhage. Five random fields from each group were semi-quantitatively scored for ALI based on the four criteria mentioned. A portion of lung tissue was frozen in liquid nitrogen for RNA extraction. The mortality study involved high-dose LPS (10 mg/kg) induction, with mice monitored twice daily for up to 7 days. Untreated normal mice were used as the Naïve group, with the main groups classified as follows: (1): Naïve; (2) LPS.

To isolate macrophages from LPS-induced mice at specified post-induction time points, bronchoalveolar lavage was performed. TR-AMs were identified by specific surface markers CD45^+^CD11c^high^SiglecF^high^MERTK^high^CD64^high^ and sorted using flow cytometry. Experiments were conducted using cell suspensions with purity ≥95%, confirmed by flow cytometry (FACS) and Pappenheim-stained cell smears. Subsequently, 50,000 cells were transferred in 50 μL sterile PBS^−/−^ via oropharyngeal instillation to Ccr2^−/−^ mice on the third-day post-induction. The main groups were categorized as follows: (1): Control (no cells); (2) TR-AMs.

For the Sbno2 expression study in TR-AMs, cells were incubated with anti-Sbno2 antibody (SBNO2 antibody (A-3) from Santa Cruz Biotechnology: sc-515634) or recommended IgG isotype control antibody at 4 °C for 10 min, followed by washing and direct transfer. The main groups were classified as follows: (1): Control; (2) IgG isotype; (3) anti-Sbno2.

To study the role of AMs in LPS induction, we depleted lung AMs in C57BL/6 mice by intranasal administration of 100 μL of chlorhexidine-liposome (CL_2_MBP) (Yeasen, Shanghai, China) 2 days prior to LPS induction. BAL cell staining confirmed AM depletion, consistent with previous reports [23], which showed that intranasally administered CL_2_MBP depleted 90% of AMs compared to PBS-treated control mice. The main groups were divided as follows: (1) Wild type (WT); (2) WT-CL_2_MBP; (3) Sbno2-KO + CL_2_MBP.

For exploring the therapeutic role of Sbno2, intratracheal administration of rSbno2 (5 μg, MBS1370624, MyBioSource, USA) was conducted after lipopolysaccharide (LPS) induction for 4 h. The main groups were categorized as follows: (1): PBS; and (2) rSbno2 [14]. We chose days 3 and 7 as time points to capture the later stage of inflammation and the initiation of the repair process.

### Lung wet/dry ratio

Lung samples were weighed and then placed in a 60 °C incubator for 48 h until a constant weight was achieved to calculate the lung wet-to-dry weight ratio (W/D) [24].

### Single-cell transcriptome data acquisition

On day 7 post-modeling, one mouse from each Naïve and LPS group was randomly selected, and lung tissue was collected, minced, and digested using a lung dissociation kit (130-095-927, Miltenyi Biotech, Shanghai, China) combined with genetics dissociation reagent (130-093-235, Miltenyi Biotech) following the manufacturer’s instructions. After digestion at 37 °C with agitation for 30 min, the cell suspension was filtered through a 70 μm cell strainer, centrifuged at 300 × *g* for 5 min, and red blood cells were lysed using RBC lysis buffer. After centrifugation, cell counting, and viability assessment, the cells were resuspended at a concentration of 1 × 10^6 ^mL^−^^1^ for subsequent analysis. All samples were processed within 2 h post-collection in a Biosafety Level 3 (BSL-3) laboratory. Cell sorting in the cell suspension from lung tissue was performed using the LSRFortessa™ Cell Analyzer (BD Bioscience, USA). RNA was purified and libraries were prepared with the 10x v2 5’ Gene Expression Library Kit according to the instructions. Base calling was conducted using RTA2 software, and fastq files were generated with Cellranger fast (v3.0.2), followed by alignment to the GRCm38 reference genome provided by 10X Genomics using Cellranger. The expression matrix generated by Cellranger was loaded into the Seurat package (v4.0.2) in R for downstream analysis, including t-distributed stochastic neighbor embedding (t-SNE) analysis [25].

### Single-cell analysis

Data quality control was conducted using the criteria nFeature_RNA > 200, nCount_RNA < 100,000, and percent.mt <20. To reduce the dimensionality of the scRNA-Seq dataset, principal component analysis (PCA) was performed based on the top 2000 highly variable genes. The top 17 principal components were selected for downstream analysis using the Elbowplot function in the Seurat package. The FindClusters function in Seurat was utilized to identify main cell subgroups with a resolution set to default (res = 0.5). Subsequently, the t-SNE algorithm was applied for nonlinear dimensionality reduction of the scRNA-Seq sequencing data. Marker genes for various cell subgroups were screened using the Seurat package. Cell annotation was performed by identifying known cell lineage-specific marker genes and utilizing the CellMarker online platform. Pseudotime analysis was conducted using the “monocle” package [26], cell-cell interaction analysis was carried out using the “cellchat” package, and enrichment analysis was performed using the “ClusterGVis” package. Cell subtypes were named based on their highly expressed features.

### Transcriptome sequencing

On day 7 post-modeling, BALF was collected from mice in the Naïve (*n* = 4) and LPS groups (*n* = 4) for sorting of TR-AMs, which were then subjected to transcriptome sequencing. Total RNA was extracted from the cells using TRIzol (Catalog number: 15596026, ThermoFisher, USA) and the purity and concentration of the extracted RNA were assessed using a nanodrop2000 spectrophotometer (ThermoFisher, USA). Following the instructions of the PrimeScript RT reagent Kit (RR047A, Takara, Japan), the RNA was reverse transcribed into cDNA for transcriptome sequencing. Differential analysis was conducted using the R package “limma,” with |log2(FoldChange)| > 1 and a significant *P* value < 0.05 as the criteria for selecting differentially expressed genes, thus obtaining the differentially expressed genes [27].

### Flow cytometry and cell sorting

Cells (1–5 × 10^5^) obtained from bronchoalveolar lavage or from lavaged, perfused and homogenized mouse lungs were resuspended in FACS buffer (PBS^−/−^, 10% FBS, 0.1% NaN_3_), pre-incubated with Fc block for 5 min, and stained with fluorescently labeled antibodies at 4 °C for 30 min. Antibodies used for FACS analysis of lung epithelial cells, macrophage subtypes/phenotypes, AEC proliferation and apoptosis, and other fluorescence markers are listed in Table S3. Cell viability staining was performed using 7-AAD (BioLegend Cat No. 420403) (1/100) or Sytox (Thermo Fisher Scientific, Cat No. S34862) (1/1000) to exclude dead cells. Unless specified, all fluorescently labeled antibodies were sourced from BioLegend. Cells were fixed overnight in 1% paraformaldehyde and analyzed the next day using the LSRFortessa™ cell analyzer (BD Bioscience, USA). Flowjo software (Ashland, OR, USA) was used to analyze samples, and detailed antibody information for flow cytometry is provided in Table S3 [28, 29].

### Isolation of mouse AECs

The lungs of mice were initially perfused with Hank’s Balanced Salt Solution (HBSS, Catalog No. 14175095, Gibco™, Thermo Fisher, USA) and then incubated in an environment containing dispase (Catalog No. 17105041, Gibco™, Thermo Fisher, USA) via tracheal instillation for 40 min. After removal of the trachea and proximal bronchial tree, the lungs were homogenized in Dulbecco’s Modified Eagle Medium (DMEM, GentleMACS, MACS Miltenyi Biotech) containing 2.5% HEPES and 0.01% DNase (Catalog No. 90083, Thermo Scientific™), and filtered through 100 and 40 µm nylon filters. The cell suspension was incubated for 30 min with biotinylated anti-CD45, anti-CD16/32, and anti-CD31 monoclonal antibodies (antibody details in Table S3) at 37 °C to remove leukocytes and endothelial cells using biotin-coated magnetic beads and magnetic separation techniques for further cultivation. AECs with a purity of ≥90%, as confirmed by flow cytometry, were seeded at a density of 2.5 × 10^5^ cells per square centimeter on 4 µm pore size Transwell culture inserts (Corning Inc.) in 24-well plates or plated on culture slides and cultured in DMEM (GentleMACS, MACS Miltenyi Biotec) containing HEPES, L-glutamine, fetal bovine serum, and penicillin/streptomycin to obtain AECII cells, with AECII cell proliferation and differentiation leading to an AECI-like phenotype (AEC) [30].

### In vitro analysis of AECs

AECs were induced with LPS at a multiplicity of infection (MOI) of 0.5. In the co-culture experiment, AECs were initially seeded and allowed to reach confluence before LPS induction. Following induction for 24 h, TR-AMs were flow-sorted from the BALF of Naïve and LPS-induced mice and directly added to the monolayer cells for a 24-h co-culture period. Subsequently, Annexin V staining was used for apoptosis assessment, Ki67 for proliferation experiments, and quantitative analysis was performed using Flow Cytometry (FACS). For proliferation experiments, mouse AECs were cultured in DMEM supplemented with penicillin/streptomycin, L-glutamine, and 2% FBS. After incubating the cells at 37 °C for 4 days, they were treated with rSbno2 (40 ng/mL, Catalog No. MBS1370624, MyBioSource, USA) for 12 h. For qPCR analysis, mouse AECs were cultured in DMEM supplemented with penicillin/streptomycin, L-glutamine, and 10% FBS, induced with LPS upon reaching confluence, and treated with rSbno2 (20–40 ng/mL) for 12 h. Cells were preserved in RLT buffer, stored at −80 °C, and kept for subsequent qPCR analysis. In experiments stimulating TR-AM with AEC supernatant, AECs were cultured following the aforementioned method. When cells reached confluence, LPS was induced as described above and incubated at 37 °C for 12/24 h. The supernatant from these cells was used to stimulate TR-AM (seeded at a density of 3 × 10^5^/cm^2^ in a 24-well plate and cultured in DMEM supplemented with penicillin/streptomycin, L-glutamine) [31].

### Detection of AEC apoptosis

The AEC pellet was first resuspended in 10 µL of FC blocking buffer. After 15 min, 100 µL of Annexin V buffer solution (No. 640922, BioLegend, USA) containing FITC-labeled Annexin V antibody (dilution ratio of 1:20) was added. Subsequently, the samples were incubated at 4 °C for 15 to 25 min. All stained cell suspensions were transferred to 5 mL polystyrene tubes and analyzed using the BD LSRFortessa™ flow cytometer to quantitatively determine the proportion of apoptotic AECs [31].

### Detection of AEC proliferation

The precipitate of AEC II was resuspended in 150 µL of diluted permeabilization/fixation buffer (Catalog No. 88-8824-00, eBioscience™, Thermo Fisher, Shanghai, China) and incubated at room temperature for 30 min. Following incubation, 100 µL of permeabilization/wash buffer was added to the samples, followed by centrifugation at 1000 × *g* for 5 min at 4 °C. After removing the supernatant, 50 µL of Ki67 antibody (diluted 1:50 in permeabilization/wash buffer) was added to each sample and incubated in the dark at 4 °C for 1 h. Upon completion of staining, cells were washed with permeabilization/wash buffer and centrifuged again at 1000 × *g* for 5 min. Finally, the cell pellet was resuspended in 200 µL of sorting buffer for quantitative analysis of cell proliferation [31].

### MTT assay

The MTT assay was used to analyze cell viability: 0.3 × 10^4^ cells were seeded in each well of a 96-well plate, treated after overnight culture, and co-cultured for 24 h. Then, 10 μL of MTT solution (5 mg/mL in PBS, 11465007001, Merck) was added to each well and incubated at 37 °C for 4 h. The resulting crystals were dissolved in 150 μL of dimethyl sulfoxide (DMSO; Sigma-Aldrich, St. Louis, Missouri, USA), and absorbance was measured at 550 nm [32].

### Measurement of pulmonary alveolar leakage

The assessment of pulmonary alveolar barrier leakage was conducted by intravenously injecting 100 µL of FITC-labeled albumin (Merck KGaA, Darmstadt, Germany). Subsequently, the fluorescence ratio of FITC in BALF and serum (serum diluted 1:100) was quantified using a fluorescence reader (FLX 800, Bio-Tek Instruments, USA). The data were processed as per the previously outlined method and reported in arbitrary units (AU) [31].

### Experimental lung organoids

Bronchoalveolar Lung Organoids (BALO) were generated by co-culturing flow cytometry-sorted EpCAM^high^Sca-1^+^CD24^low^ mouse lung epithelial stem/progenitor cells with EpCAM-Sca-1^+^ lung resident mesenchymal cells, as described in a previous study [33]. In the lung organoid culture, 3 × 10+ wild-type TR-AMs were co-cultured with the organoids in Matrigel® (Corning) supplemented with α-MEM, 10% FBS, 100 U/mL penicillin, 0.1 mg/mL streptomycin, 2 mM L-glutamine, 1× insulin/transferrin/selenium, and 0.0002% heparin. For selected experiments, an anti-Sbno2 antibody (20 ng/mL, HPA041867, Atlas Antibodies, Sweden) or isotype control (20 ng/mL) was added to the organoid culture medium at cell seeding. The quantification and size assessment of BALO were manually analyzed via optical microscopy, observed using the EVOS M5000 imaging system (AMF7000, Thermo Fisher Scientific), with data reported as the mean per well, where individual data points represent a single well [33]. The HPA041867 antibody used for Sbno2 blockade in the organoid experiments is reported to have cross-reactivity with mouse Sbno2, and future species-specific validation would further enhance the robustness of the data.

### Western blot

TR-AMs sorted from mouse BALF were lysed in RIPA buffer (P0013B, Beyotime Biotechnology, Shanghai, China) containing 1% protease and phosphatase inhibitors. Total protein concentrations were quantified using the BCA Protein Assay Kit (A53226, Thermo Fisher Scientific, Rockford, IL, USA). Proteins were separated via SDS-PAGE and transferred onto PVDF membranes (IPVH85R, Millipore, MA, USA) using a wet transfer method. Membranes were blocked with 5% BSA at room temperature for 1 h and incubated overnight at 4°C with the following primary antibodies: anti-Sbno2 (1:1000, Santa Cruz Biotechnology, SBNO2 (A-3), sc-515634) and GAPDH (ab181602, 1:10,000, Abcam, Cambridge, UK). After washing, membranes were incubated with HRP-conjugated secondary antibody IgG (ab6721, 1:5000, Abcam, UK) for 2 h. Membranes were washed three times with TBST (5 min each), and detection was performed using a chemiluminescence imaging system. Protein expression levels were quantified using ImageJ software (V1.48, National Institutes of Health, USA) and normalized to GAPDH as a loading control [34]. Each experiment was repeated three times. All the Full and uncropped western blots can be found at the Supplemental Materials.

### Enzyme-linked immunosorbent assay (ELISA)

The levels of Sbno2 in mouse BALF were measured using the ELISA kit (abx544819, Abbexa, UK) according to the manufacturer’s protocol. Absorbance was detected at 450 nm using the Epoch microplate reader (BioTek, USA) [23].

### Immunofluorescence staining

Lung tissue samples were fixed in 4% paraformaldehyde, dehydrated, cleared, embedded in paraffin, and sectioned for histological analysis. For immunofluorescence staining, the tissue sections were deparaffinized, rehydrated, and blocked with 2% BSA before proceeding with the experimental steps. Primary antibodies were diluted according to the manufacturer’s instructions (antibody details provided in Table S3) and then incubated at 4 °C overnight. After 24 h, the sections were washed with PBS^−/−^, and incubated with donkey anti-rabbit secondary antibody (A-11008, Thermo Fisher, 1:500, USA) at room temperature for 1 h. Following PBS^−/−^ rinses, the nuclei were stained with DAPI (C1002, Beyotime, China) for 5 min, and excess DAPI was removed by washing the slides with PBS three times for 5 min each. The stained sections were observed and captured under a fluorescence microscope (FV-1000/ES, Olympus, Japan) for analysis, with quantification based on the fluorescence coverage area under a fixed field of view at 40× magnification, averaging results from 6 fields per group.

For organoid staining, the cultures were fixed in 4% paraformaldehyde for 15 min, washed with PBS, and then stained with LipidTOX™ neutral lipid dye solution (H34477, Thermo Scientific) for 6 h to visualize the alveoli. Following PBS rinses, the cultures were stained with DAPI (C1002, Beyotime, China) and mounted for imaging. Confocal images of the organoids were obtained using a Leica SP5 confocal microscope (software version LAS AF 2.7.3). Quantification of the organoids (area, diameter, and alveolar count) involved manually outlining each organoid in Fiji and manually marking each alveolus on the confocal z-stack images obtained for each organoid [35].

### TUNEL immunofluorescence staining

Lung tissue samples were fixed in 4% paraformaldehyde, dehydrated, cleared, embedded in paraffin, and sectioned. The sections were treated with proteinase K solution to remove protein coating. The TUNEL reagent kit (C1089, Beyotime Biotechnology Co., Ltd.) was prepared following the manufacturer’s instructions, and TUNEL working solution was added to the samples, avoiding bubbles. Samples were incubated in a dark, humidified chamber at 37 °C for 2 h. After the TUNEL reaction, T-1α antibody (PA5-23405, ThermoFisher) was applied to the sample, also avoiding bubbles. After 1 h of incubation in the dark at 37 °C, samples were washed three times with PBS, and Goat Anti-Rabbit IgG H&L (Alexa Fluor® 488) secondary antibody (ab150077, Abcam) was applied for 2 h. Samples were washed with appropriate buffer to remove excess antibody, stained with DAPI (C1002, Beyotime, China), and mounted for imaging. Samples were observed under a fluorescence microscope to detect TUNEL and T-1α labeled cells and alveolar epithelial progenitor cells [36].

### RT-qPCR

According to the manufacturer’s instructions, total RNA was extracted using Trizol reagent (15596026, Invitrogen, USA), and then reverse transcribed into cDNA using the PrimeScript RT reagent Kit (RR047A, Takara, Japan). The synthesized cDNA was analyzed by RT-qPCR using the Fast SYBR Green PCR kit (11736059, Thermo Fisher Scientific (China) Co., Ltd, Shanghai, China) with 3 replicates per well. The primers for each gene were synthesized by TaKaRa (details in Table S4), with Gapdh serving as the reference gene. The relative expression levels of each gene were analyzed using the 2^−ΔΔCt^ method, where ΔΔCt = (average Ct value of target gene in experimental group - average Ct value of housekeeping gene in experimental group) − (average Ct value of target gene in control group − average Ct value of housekeeping gene in control group). All RT-qPCR analyses were performed in triplicate [37–39].

### Statistical analysis

The data were derived from at least three independent experiments and are presented as mean ± standard deviation (Mean ± SD). For comparisons between two groups, two-sample independent t-tests were performed. For comparisons involving three or more groups, a one-way analysis of variance (ANOVA) was utilized. Following a significant result in the analysis of variance, Tukey’s HSD post hoc test was conducted to compare differences between each group. In cases of non-normal distribution or heteroscedasticity, the Mann–Whitney U test or Kruskal–Wallis H test was employed.

All statistical analyses were carried out using GraphPad Prism 9 (GraphPad Software, Inc.) and R programming language. The significance level for all tests was set at 0.05, with a two-tailed p-value less than 0.05 considered statistically significant [40].

## Supplementary information

Table S1 diff_AMs.markers

Table S2 diff_Epithelial_cells.markers

supplementary figures and tables

Full and uncropped western blots of figure 6A-1

Full and uncropped western blots of figure 6A-2

## Data Availability

All data can be provided as needed.

## References

[1] Hendrickson CM, Matthay MA. Endothelial biomarkers in human sepsis: pathogenesis and prognosis for ARDS. Pulm Circ. 2018;8:1–12. 10.1177/2045894018769876 PMC5912282 29575977

[2] Selickman J, Vrettou CS, Mentzelopoulos SD, Marini JJ. COVID-19-related ARDS: key mechanistic features and treatments. JCM. 2022;11:4896. 36013135 10.3390/jcm11164896 PMC9410336

[3] Weidenfeld S, Chupin C, Langner DI, Zetoun T, Rozowsky S, Kuebler WM. Sodium-coupled neutral amino acid transporter SNAT2 counteracts cardiogenic pulmonary edema by driving alveolar fluid clearance. Am J Physiol Lung Cell Mol Physiol. 2021;320:L486–L497. 33439101 10.1152/ajplung.00461.2020

[4] Cao J, Ding C, Huang J, Chen Y, Chen Y. Pulmonary vascular endothelial glycocalyx degradation contributes to acute lung injury in experiencing heatstroke. Shock. 2023;59:966–72. 37040184 10.1097/SHK.0000000000002130

[5] Yu J, Xu C, Lee JS, Alder JK, Wen Z, Wang G, et al. Rapid postmortem ventilation improves donor lung viability by extending the tolerable warm ischemic time after cardiac death in mice. Am J Physiol Lung Cell Mol Physiol. 2021;321:L653–L662. 34318693 10.1152/ajplung.00011.2021

[6] Aegerter H, Lambrecht BN, Jakubzick CV. Biology of lung macrophages in health and disease. Immunity. 2022;55:1564–80. 36103853 10.1016/j.immuni.2022.08.010 PMC9533769

[7] Hill W, Lim EL, Weeden CE, Lee C, Augustine M, Chen K, et al. Lung adenocarcinoma promotion by air pollutants. Nature. 2023;616:159–67. 37020004 10.1038/s41586-023-05874-3 PMC7614604

[8] Bo H, Moure UAE, Yang Y, Pan J, Li L, Wang M, et al. Mycobacterium tuberculosis-macrophage interaction: Molecular updates. Front. Cell. Infect. Microbiol. 2023;13. 10.3389/fcimb.2023.1062963. 10.3389/fcimb.2023.1062963 PMC10020944 36936766

[9] Feng Z, Jing Z, Li Q, Chu L, Jiang Y, Zhang X, et al. Exosomal STIMATE derived from type II alveolar epithelial cells controls metabolic reprogramming of tissue-resident alveolar macrophages. Theranostics. 2023;13:991–1009. 36793853 10.7150/thno.82552 PMC9925314

[10] Chang C-Y, Armstrong D, Corry DB, Kheradmand F. Alveolar macrophages in lung cancer: opportunities and challenges. Front. Immunol. 2023;14. 10.3389/fimmu.2023.1268939. 10.3389/fimmu.2023.1268939 PMC10562548 37822933

[11] Hou F, Wang H, Zheng K, Yang W, Xiao K, Rong Z, et al. Distinct transcriptional and functional differences of lung resident and monocyte-derived alveolar macrophages during the recovery period of acute lung injury. Immune Netw. 2023;23. 10.4110/in.2023.23.e24. 10.4110/in.2023.23.e24 PMC10320419 37416929

[12] Syme TE, Grill M, Hayashida E, Viengkhou B, Campbell IL, Hofer MJ. Strawberry notch homolog 2 regulates the response to interleukin-6 in the central nervous system. J Neuroinflammation. 2022;19. 10.1186/s12974-022-02475-1. 10.1186/s12974-022-02475-1 PMC9145108 35624480

[13] Aschenbrenner D, Nassiri I, Venkateswaran S, Pandey S, Page M, Drowley L, et al. An isoform quantitative trait locus in SBNO2 links genetic susceptibility to Crohn’s disease with defective antimicrobial activity. Nat Commun. 2024;15. 10.1038/s41467-024-47218-3. 10.1038/s41467-024-47218-3 PMC11133462 38806456

[14] El Kasmi KC, Smith AM, Williams L, Neale G, Panopolous A, Watowich SS, et al. Cutting edge: a transcriptional repressor and corepressor induced by the STAT3-regulated anti-inflammatory signaling pathway. J Immunol. 2007;179:7215–9. 18025162 10.4049/jimmunol.179.11.7215

[15] Woo YD, Jeong D, Chung DH. Development and functions of alveolar macrophages. Mol Cells. 2021;44:292–300. 33972474 10.14348/molcells.2021.0058 PMC8175155

[16] Hung L-Y, Sen D, Oniskey TK, Katzen J, Cohen NA, Vaughan AE, et al. Macrophages promote epithelial proliferation following infectious and non-infectious lung injury through a Trefoil factor 2-dependent mechanism. Mucosal Immunol. 2019;12:64–76. 30337651 10.1038/s41385-018-0096-2 PMC6301101

[17] Hewitt RJ, Lloyd CM. Regulation of immune responses by the airway epithelial cell landscape. Nat Rev Immunol. 2021;21:347–62. 33442032 10.1038/s41577-020-00477-9 PMC7804588

[18] Cheng P, Li S, Chen H. Macrophages in lung injury, repair, and fibrosis. Cells. 2021;10:436. 33670759 10.3390/cells10020436 PMC7923175

[19] Roque W, Romero F. Cellular metabolomics of pulmonary fibrosis, from amino acids to lipids. Am J Physiol Cell Physiol. 2021;320:C689–C695. 33471621 10.1152/ajpcell.00586.2020 PMC8163573

[20] Moss BJ, Ryter SW, Rosas IO. Pathogenic mechanisms underlying idiopathic pulmonary fibrosis. Annu Rev Pathol Mech Dis. 2022;17:515–46. 10.1146/annurev-pathol-042320-030240 34813355

[21] Tanner L, Single AB, Bhongir RKV, Heusel M, Mohanty T, Karlsson CAQ, et al. Small-molecule-mediated OGG1 inhibition attenuates pulmonary inflammation and lung fibrosis in a murine lung fibrosis model. Nat Commun. 2023;14. 10.1038/s41467-023-36314-5. 10.1038/s41467-023-36314-5 PMC9902543 36746968

[22] Maruyama K, Uematsu S, Kondo T, Takeuchi O, Martino MM, Kawasaki T, et al. Strawberry notch homologue 2 regulates osteoclast fusion by enhancing the expression of DC-STAMP. J Exp Med. 2013;210:1947–60. 23980096 10.1084/jem.20130512 PMC3782043

[23] Xia L, Zhang C, Lv N, Liang Z, Ma T, Cheng H, et al. AdMSC-derived exosomes alleviate acute lung injury via transferring mitochondrial component to improve homeostasis of alveolar macrophages. Theranostics. 2022;12:2928–47. 35401830 10.7150/thno.69533 PMC8965475

[24] Zhan Z, Lian Z, Bai H. Dexamethasone inhibited angiotensin II and its receptors to reduce sepsis-induced lung and kidney injury in rats. PLoS ONE. 2024;19:e0308557. 39178201 10.1371/journal.pone.0308557 PMC11343412

[25] Schyns J, Bai Q, Ruscitti C, Radermecker C, De Schepper S, Chakarov S, et al. Non-classical tissue monocytes and two functionally distinct populations of interstitial macrophages populate the mouse lung. Nat Commun. 2019;10. 10.1038/s41467-019-11843-0. 10.1038/s41467-019-11843-0 PMC6722135 31481690

[26] Liu W, Hu H, Shao Z, Lv X, Zhang Z, Deng X, et al. Characterizing the tumor microenvironment at the single-cell level reveals a novel immune evasion mechanism in osteosarcoma. Bone Res. 2023;11. 10.1038/s41413-022-00237-6. 10.1038/s41413-022-00237-6 PMC9810605 36596773

[27] Ergin S, Kherad N, Alagoz M. RNA sequencing and its applications in cancer and rare diseases. Mol Biol Rep. 2022;49:2325–33. 34988891 10.1007/s11033-021-06963-0 PMC8731134

[28] Song Y, Dou H, Li X, Zhao X, Li Y, Liu D, et al. Exosomal miR-146a contributes to the enhanced therapeutic efficacy of interleukin-1β-primed mesenchymal stem cells against sepsis. Stem Cells. 2017;35:1208–21. 28090688 10.1002/stem.2564

[29] Zhong C, Tao B, Yang F, Xia K, Yang X, Chen L, et al. Histone demethylase JMJD1C promotes the polarization of M1 macrophages to prevent glioma by upregulating miR-302a. Clinical & Translational Med. 2021;11. 10.1002/ctm2.424. 10.1002/ctm2.424 PMC8473479 34586733

[30] Herold S, von Wulffen W, Steinmueller M, Pleschka S, Kuziel WA, Mack M, et al. Alveolar Epithelial Cells Direct Monocyte Transepithelial Migration upon Influenza Virus Infection: Impact of Chemokines and Adhesion Molecules. J Immunol. 2006;177:1817–24. 16849492 10.4049/jimmunol.177.3.1817

[31] Pervizaj-Oruqaj L, Selvakumar B, Ferrero MR, Heiner M, Malainou C, Glaser RD, et al. Alveolar macrophage-expressed Plet1 is a driver of lung epithelial repair after viral pneumonia. Nat Commun. 2024;15. 10.1038/s41467-023-44421-6. 10.1038/s41467-023-44421-6 PMC10761876 38167746

[32] Tian R, Li H, Ren S, Li S, Fang R, Liu Y. circRNA THBS1 silencing inhibits the malignant biological behavior of cervical cancer cells via the regulation of miR-543/HMGB2 axis. Open Medicine. 2023;18. 10.1515/med-2023-0709. 10.1515/med-2023-0709 PMC10350892 37465349

[33] Vazquez-Armendariz AI, Heiner M, El Agha E, Salwig I, Hoek A, Hessler MC, et al. Multilineage murine stem cells generate complex organoids to model distal lung development and disease. The EMBO J. 2020;39. 10.15252/embj.2019103476. 10.15252/embj.2019103476 PMC7604576 32985719

[34] Wang Y, Wang C, Fu Z, Zhang S, Chen J. miR-30b-5p inhibits proliferation, invasion, and migration of papillary thyroid cancer by targeting GALNT7 via the EGFR/PI3K/AKT pathway. Cancer Cell Int. 2021;21. 10.1186/s12935-021-02323-x. 10.1186/s12935-021-02323-x PMC8611849 34819077

[35] Ma X-H, Piao S-F, Dey S, Mcafee Q, Karakousis G, Villanueva J, et al. Targeting ER stress–induced autophagy overcomes BRAF inhibitor resistance in melanoma. J Clin Invest. 2014;124:1406–17. 24569374 10.1172/JCI70454 PMC3934165

[36] Becker E Jr, Husain M, Bone N, Smith S, Morris P, Zmijewski JW. AMPK activation improves recovery from pneumonia-induced lung injury via reduction of er-stress and apoptosis in alveolar epithelial cells. Respir Res. 2023;24. 10.1186/s12931-023-02483-6. 10.1186/s12931-023-02483-6 PMC10337128 37438806

[37] Ayuk SM, Abrahamse H, Houreld NN. The role of photobiomodulation on gene expression of cell adhesion molecules in diabetic wounded fibroblasts in vitro. J Photochem Photobiol B Biol. 2016;161:368–74. 10.1016/j.jphotobiol.2016.05.027 27295416

[38] Wu Q, Yi X. Down-regulation of long noncoding RNA MALAT1 protects hippocampal neurons against excessive autophagy and apoptosis via the PI3K/Akt signaling pathway in rats with epilepsy. J Mol Neurosci. 2018;65:234–45. 29858824 10.1007/s12031-018-1093-3

[39] Mao Q, Liang X-L, Zhang C-L, Pang Y-H, Lu Y-X. LncRNA KLF3-AS1 in human mesenchymal stem cell-derived exosomes ameliorates pyroptosis of cardiomyocytes and myocardial infarction through miR-138-5p/Sirt1 axis. Stem Cell Res Ther. 2019;10. 10.1186/s13287-019-1522-4. 10.1186/s13287-019-1522-4 PMC6918658 31847890

[40] Zhang Q-F, Li J, Jiang K, Wang R, Ge J, Yang H, et al. CDK4/6 inhibition promotes immune infiltration in ovarian cancer and synergizes with PD-1 blockade in a B cell-dependent manner. Theranostics. 2020;10:10619–33. 32929370 10.7150/thno.44871 PMC7482823

