# Supplementary material for: Sbno2-mediated tissue-resident alveolar macrophages: a novel therapeutic axis for sepsis-induced acute lung injury
Source: Cell Death Discov. 2026 Jan 5;12:80. doi: 10.1038/s41420-025-02772-7 (PMC12876955; doi:10.1038/s41420-025-02772-7)
Supplement: Supplementary file 3 — supplementary figures and tables [file 41420_2025_2772_MOESM3_ESM.docx]

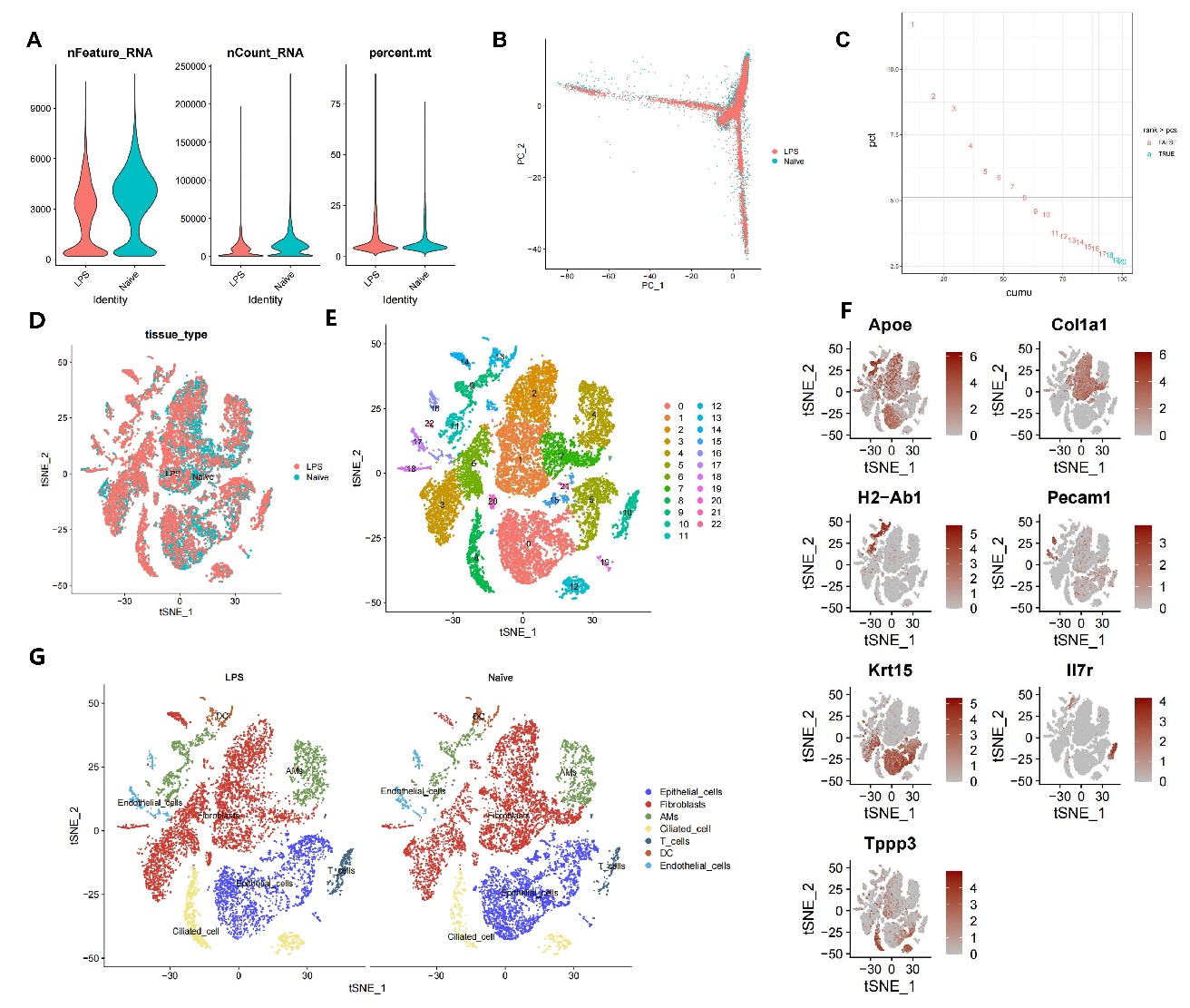


**Fig. S1. Classification of cellular components in lung tissue samples from the LPS model by single-cell RNA-seq analysis**

Note: (A) Violin plots of the number of genes per cell (nFeature_RNA), number of mRNA molecules (nCount_RNA), and percentage of mitochondrial genes (percent.mt) in the scRNA-seq data; (B) Distribution of cells in PC_1 and PC_2; each dot represents a single cell; (C) Quantitative Elbow analysis identifying the top 17 principal components for downstream analysis; (D) t-SNE clustering plots showing the distribution of cell clusters in lung tissue samples from Naïve and LPS model mice; (E) Visualization of t-SNE clustering results showing cell aggregation and distribution; each color represents a distinct cluster; (F) t-SNE plots showing the expression of marker genes for major cell types identified in this study. Red indicates high expression; gray indicates low expression; (G) t-SNE clustering plots displaying the distribution of seven cell clusters from Naïve and LPS model mouse samples.


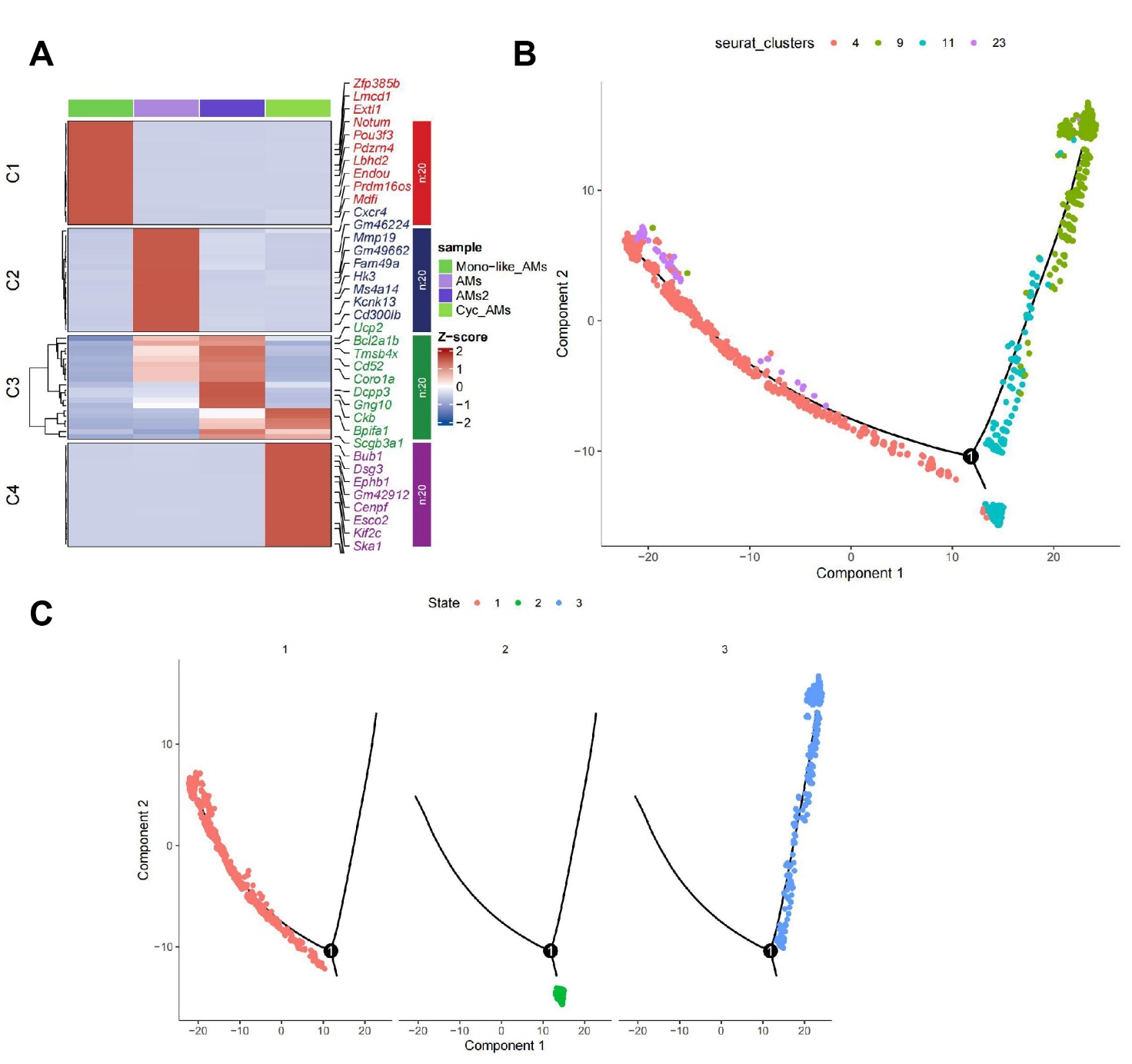


**Fig. S2. Subcluster analysis and pseudotime trajectory of AM subpopulations in LPS-induced Sepsis-ALI**

Note: (A) Heatmap of gene expression distribution across AM subpopulations; (B) Pseudotime trajectory of AM subpopulations; each dot represents a single cell, with different colors indicating different cell types; (C) Pseudotime projection of AM cells in reduced dimensional space, with color gradient representing progression along the pseudotime axis.


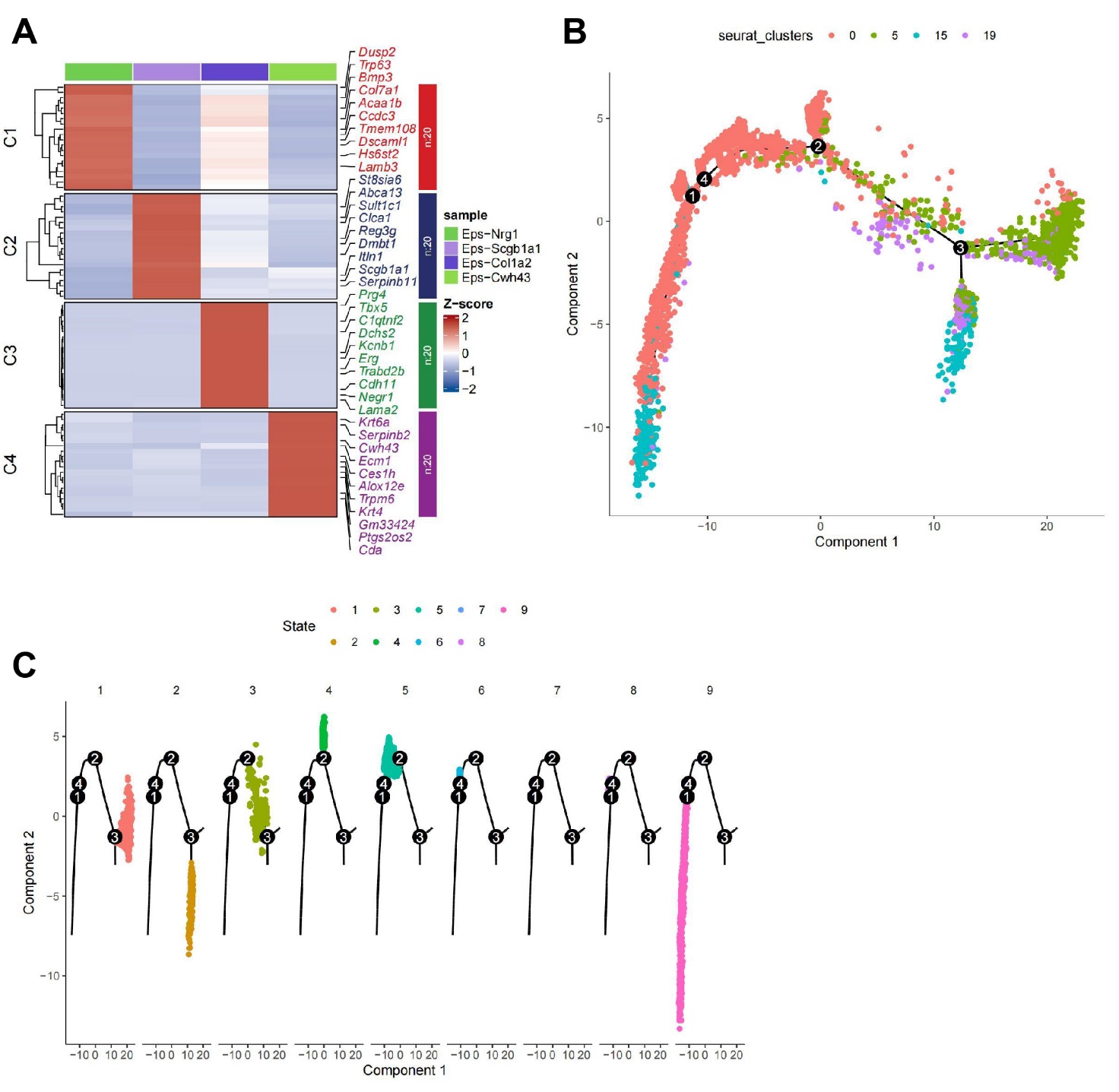


**Fig. S3. Analysis of epithelial cell subtypes and pseudo-temporal analysis in LPS-induced Sepsis-ALI.**

Note: (A) Heatmap of gene expression distribution in epithelial cell subtypes; (B) Pseudo-temporal ordering of epithelial cell subtypes, with each point representing a cell of different cell types indicated by different colors; (C) Pseudo-temporal ordering of epithelial cell subtypes in the reduced dimension space colored by pseudo-time gradient.


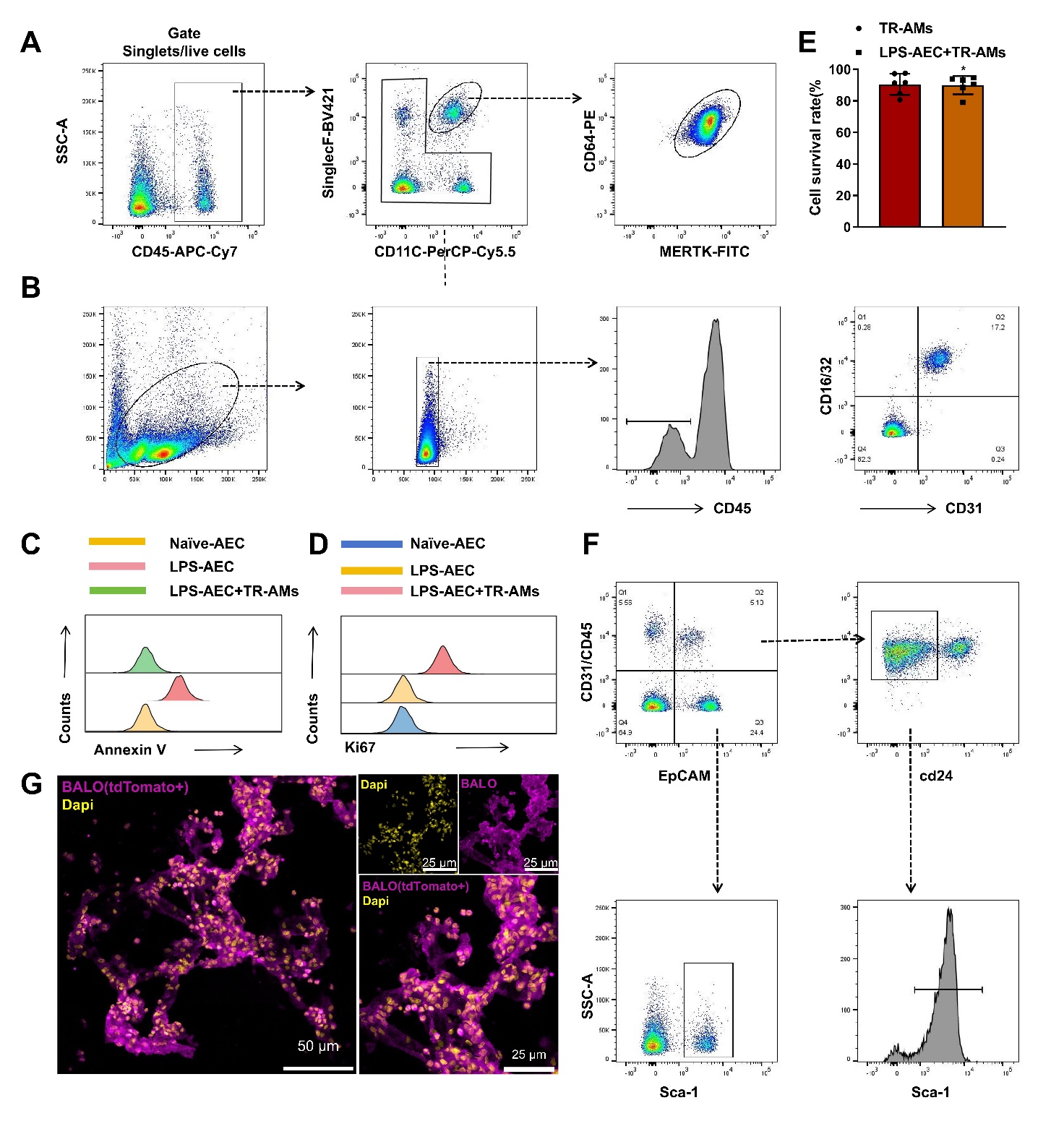


**Fig. S4. Flow cytometry gating strategy and BALO results.**

Note: (A) Representative FACS plot illustrating the gating strategy for identifying tissue-resident AMs (TR-AMs) from BALF of LPS-induced mice. TR-AMs identified by CD45^+^CD11c^high^SiglecF^high^MERTK^high^CD64^high^ characteristics; (B) Representative FACS plot illustrating the gating strategy for identifying lung epithelial cells from lung tissue of LPS-induced mice. AECs identified by CD45^+^CD31^high^CD16/32^high^ characteristics; (C) Flow cytometry analysis of TR-AMs' impact on AEC cell apoptosis; (D) Flow cytometry analysis of TR-AMs' impact on AEC cell proliferation; (E) MTT assay for TR-AM viability; (F) Gating strategy for flow sorting of EpCAM^high^Sca-1^+^CD24^low^ lung epithelial stem/progenitor cells and EpCAM+Sca-1^+^ lung resident mesenchymal cells; (G) Representative confocal images of tdTomato+ epithelial cells derived mouse BALO airway and alveolar organization (inset in left image magnified in right image; magenta, tdTomato+ epithelial cells; yellow, DAPI, scale bar represents 25 μm). *In vitro* cell experiments were repeated three times, with n=6 for *in vivo* mice; * represents *P* < 0.05.


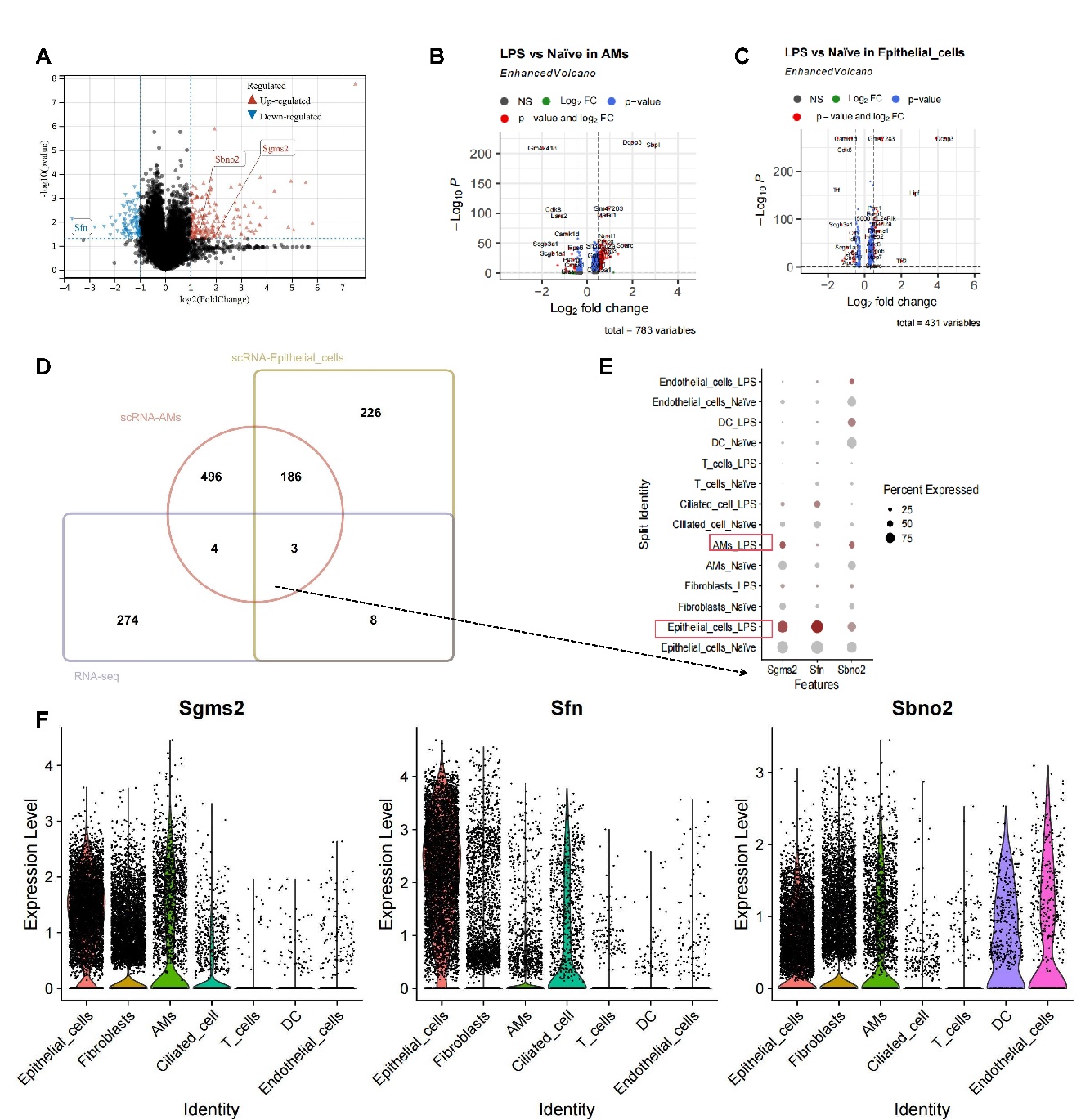


**Fig. S5. Screening of key genes mediating interactions between TR-AMs and epithelial cells.**

Note: (A) Volcano plot of differentially expressed genes between TR-AMs from LPS and Naïve groups (red: upregulated; blue: downregulated); (B) Heatmap of differentially expressed genes in AM subpopulations; (C) Heatmap of differentially expressed genes in epithelial cell subpopulations; (D) Venn diagram showing overlapping differentially expressed genes between AMs and epithelial cells (common genes: Sgms2, Sfn, Sbno2); (E) Bubble plots showing the expression of candidate genes (Sgms2, Sfn, Sbno2) across different cell subpopulations (color: expression level; size: proportion of expressing cells); (F) Violin plots showing expression levels of candidate genes.


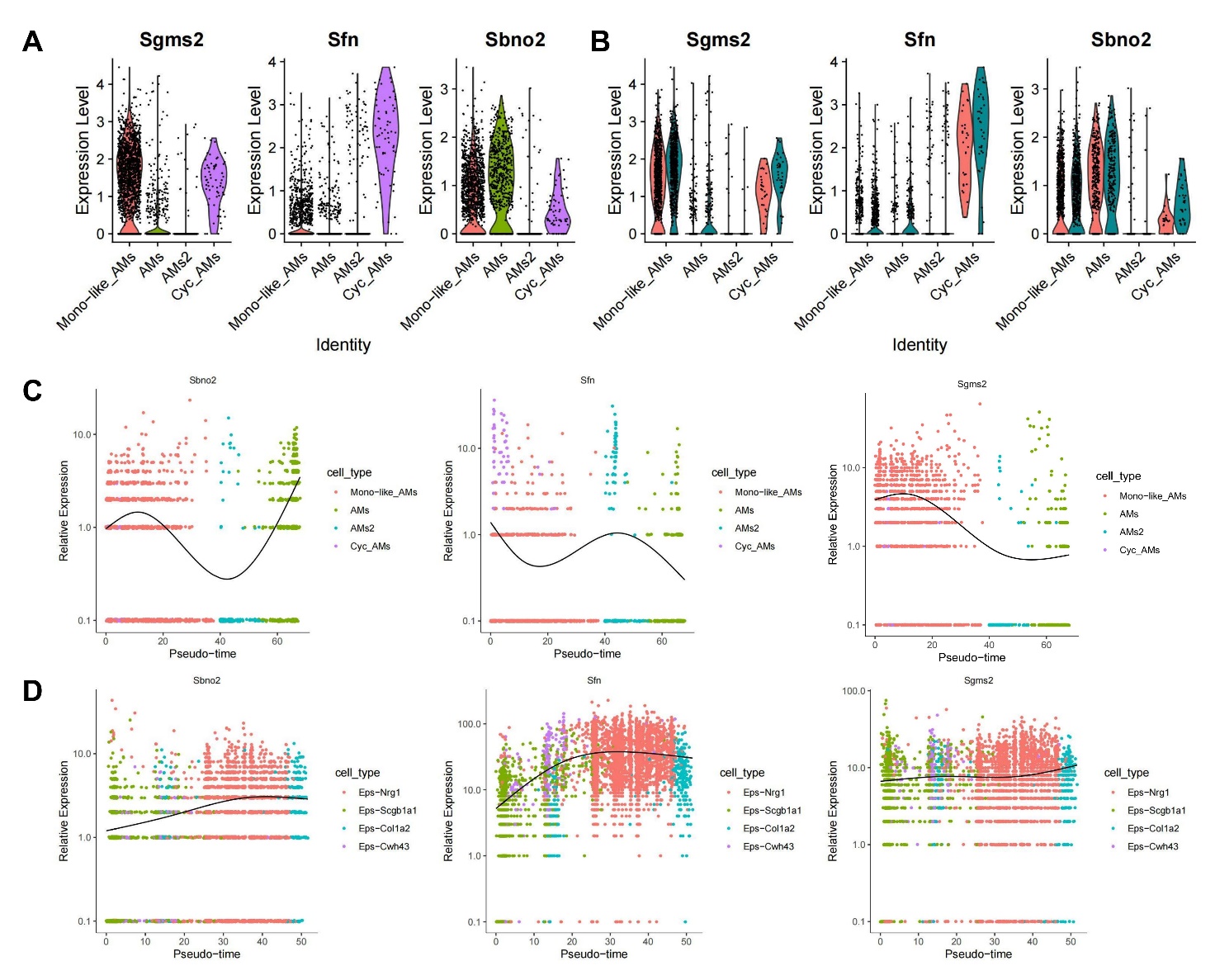


**Fig. S6. Expression of Sbno2 in AMs and epithelial cell subtypes.**

Note: (A-B) Violin plots displaying the expression levels (logarithmic) of three candidate genes in AM subtypes. The colored area represents the density distribution within each cluster. In plot B, red represents the Naïve group, and blue represents the LPS group, with data referenced from scRNA-seq experiments; (C-D) Expression changes of three candidate genes at different time points in various AMs and epithelial cell subtypes.


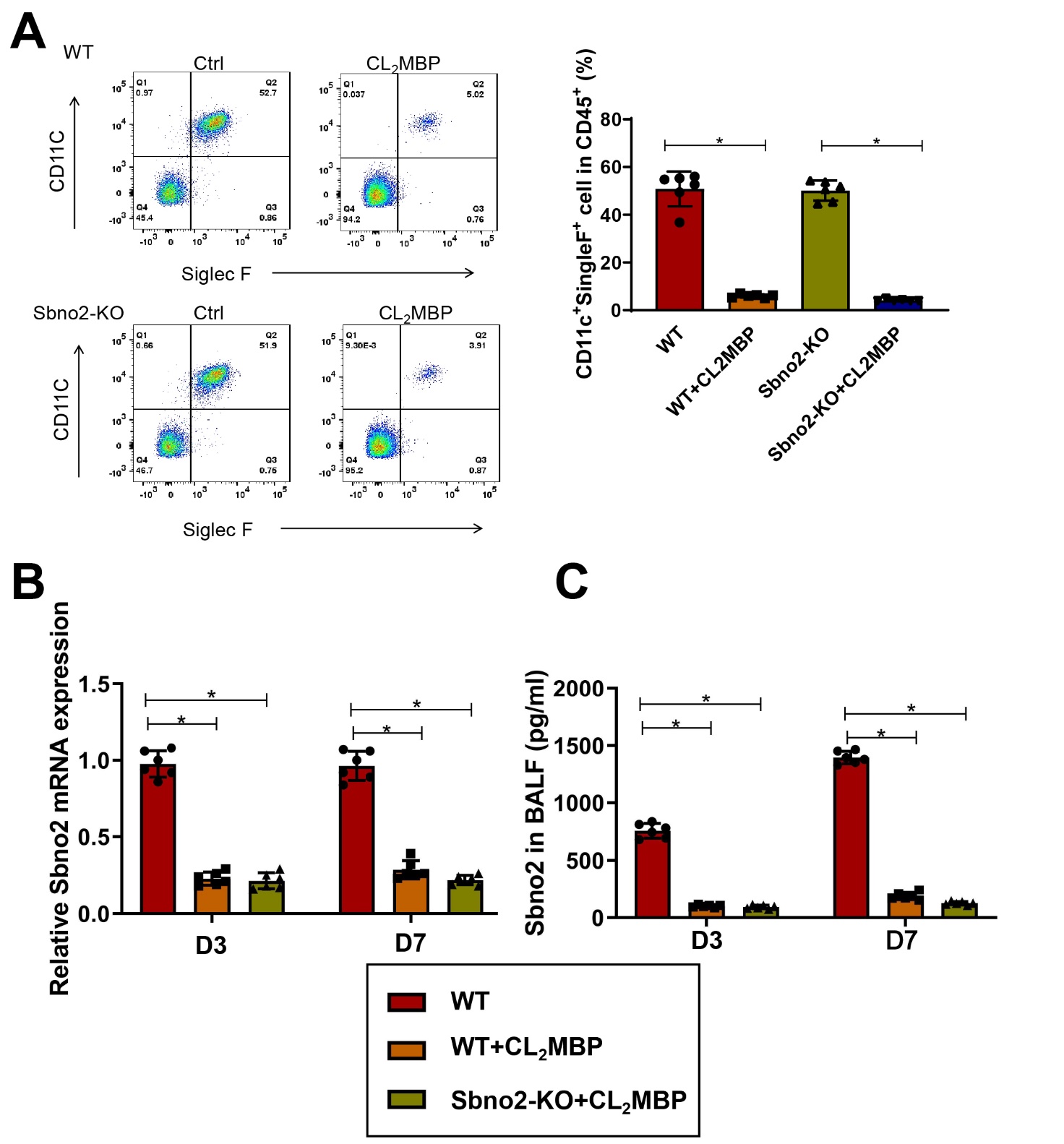


**Fig. S7. Deletion of Sbno2 in TR-AMs.**

Note: (A) Flow cytometry analysis of AMs (CD11c^+^ Siglec F^+^ CD45^+^) in BALF from different groups, representing flow cytometry plots and statistical graphs; (B) qRT-PCR to assess Sbno2 mRNA expression in BALF of Sbno2-KO versus WT mice with or without CL_2_MBP treatment; (C) ELISA to measure Sbno2 levels in BALF of Sbno2-KO versus WT mice with or without CL2MBP treatment. n=6 for *in vivo* mice; * indicates *P* < 0.05.

**Table S3. Antibody Information**

| Antibody | Manufacturer | Item number | Application |
| --- | --- | --- | --- |
| Rabbit anti-ZO-1 | abcam | ab221547 | IF |
| anti-CD326 (EpCam)  (APC-Cy7 ) | BioLegend | No 118218 | FC |
| anti-CD24 (PE-Cy7) | BioLegend | No 101821 | FC |
| anti-CD31 (Alexa Fluor 488) | BioLegend | No 102513 | FC |
| anti-CD45 (FITC) | BioLegend | No 103107 | FC |
| anti-T1α/podoplanin (APC) | BioLegend | No 127409 | FC |
| anti-Ki67(Alexa Fluor® 647) | BioLegend | No 350510 | FC |
| anti-CD16/32(FITC) | BioLegend | No 101306 | FC |
| anti-CD11c(Pacific Blue™) | BioLegend | No 337212 | FC |
| anti-SiglecF(KIRAVIA Blue 520™) | BioLegend | No 155522 | FC |
| anti-CD64(APC) | BioLegend | No 164409 | FC |
| anti-MERTK(PerCP/Cy5.5) | BioLegend | No 367622 | FC |
| anti-CD45(APC-Cy7) | BioLegend | No 103115 | FC |
| anti-Sca-1(PerCP) | BioLegend | No 108121 | FC |

Note: IF: Immunofluorescence; FC: flow cytometry.

**Table S4. RT-qPCR Primer Sequence**

| Gene | Sequences |
| --- | --- |
| Sbno2 | Forward: 5’-CATCCAGCTACAGAACCGACT-3’  Reverse: 5’-GAACCCACGAACTGTTGGTTT-3’ |
| Tjp-1 | Forward: 5’-GAGCAGGCTTTGGAGGAGAC-3’  Reverse: 5’-AGCTGCTGAACAGCAAAAGC-3’ |
| Cldn1 | Forward: 5’-CTTGGATTCTTCGGTTTGGTTGG-3’  Reverse: 5’-CTGCCGATGAAAGCTGACAC-3’ |
| Ocln | Forward: 5’-TGAAAGTCCACCTCCTTACAGA-3’  Reverse: 5’-CCGGATAAAAAGAGTACGCTGG-3’ |
| Gapdh | Forword: 5'-CCCTTAAGAGGGATGCTGCC-3' |
|  | Reverse: 5'-TACGGCCAAATCCGTTCACA-3' |
