# Supplementary figures and images for: Sbno2-mediated tissue-resident alveolar macrophages: a novel therapeutic axis for sepsis-induced acute lung injury
Source: Cell Death Discov. 2026 Jan 5;12:80. doi: 10.1038/s41420-025-02772-7 (PMC12876955; doi:10.1038/s41420-025-02772-7)

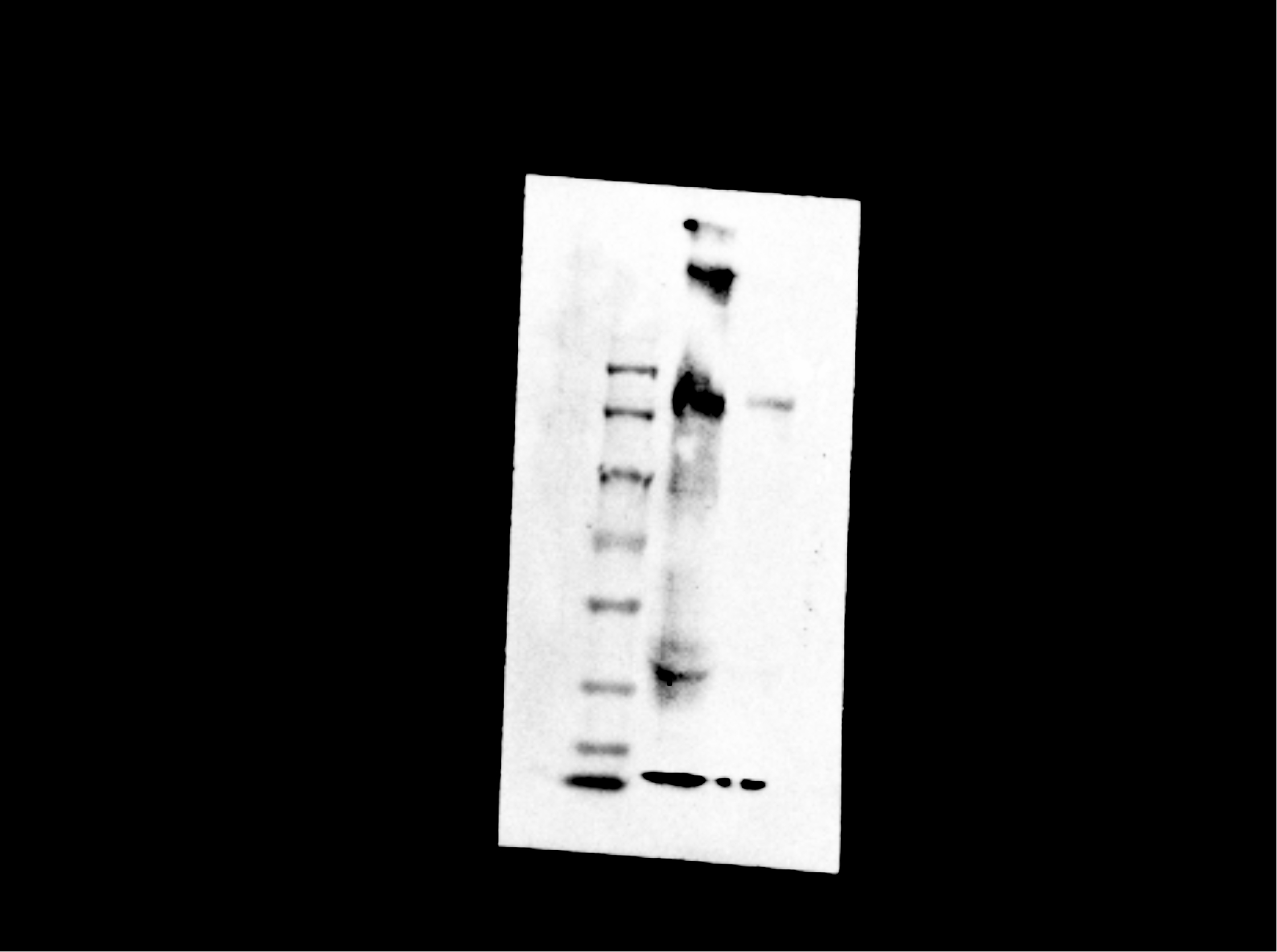

Supplement: Supplementary file 4 — Full and uncropped western blots of figure 6A-1 [file 41420_2025_2772_MOESM4_ESM.jpg]

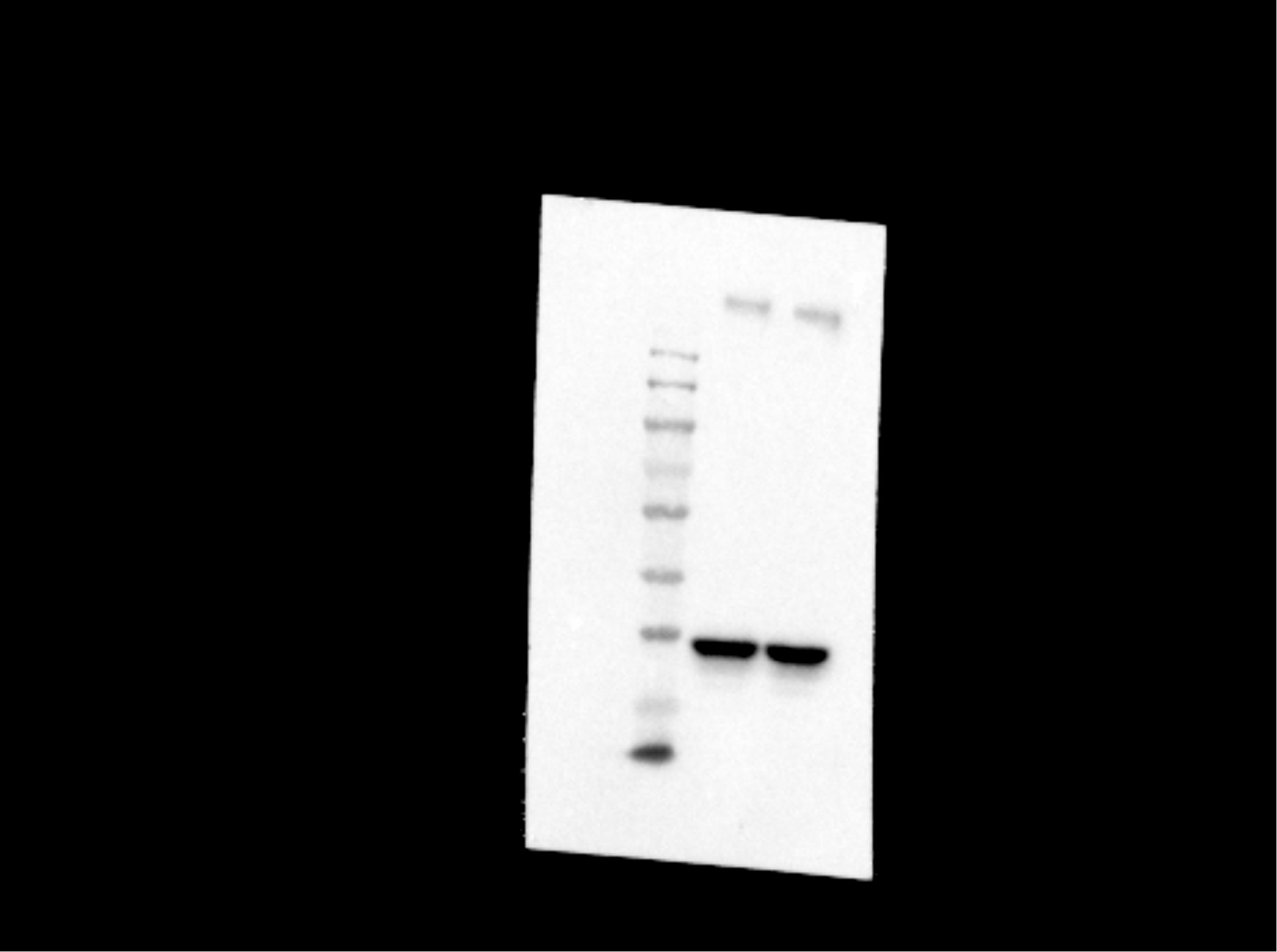

Supplement: Supplementary file 5 — Full and uncropped western blots of figure 6A-2 [file 41420_2025_2772_MOESM5_ESM.jpg]
